# Folylpolyglutamate synthetase mRNA G-quadruplexes regulate its cell protrusion localization and enhance a cancer cell invasive phenotype upon folate repletion

**DOI:** 10.1186/s12915-023-01525-1

**Published:** 2023-02-01

**Authors:** Michal Stark, May Levin, Igor Ulitsky, Yehuda G. Assaraf

**Affiliations:** 1 The Fred Wyszkowski Cancer Research Laboratory, Department of Biology, Technion-Israel Institute of Technology, 3200003 Haifa, Israel; 2 Present address: May Levin, MeMed Diagnostics Ltd, Tirat Carmel, Israel; 3 Department of Immunology and Regenerative Biology and Department of Molecular Neuroscience, Weizmann Institute of Science, 7610001 Rehovot, Israel

**Keywords:** Folylpolyglutamate synthetase, 3′UTR, G-quadruplex, Folic acid, RNA transport, Cell protrusions, Invasion

## Abstract

**Background:**

Folates are crucial for the biosynthesis of nucleotides and amino acids, essential for cell proliferation and development. Folate deficiency induces DNA damage, developmental defects, and tumorigenicity. The obligatory enzyme folylpolyglutamate synthetase (FPGS) mediates intracellular folate retention via cytosolic and mitochondrial folate polyglutamylation. Our previous paper demonstrated the association of the cytosolic FPGS (cFPGS) with the cytoskeleton and various cell protrusion proteins. Based on these recent findings, the aim of the current study was to investigate the potential role of cFPGS at cell protrusions.

**Results:**

Here we uncovered a central role for two G-quadruplex (GQ) motifs in the 3′UTR of FPGS mediating the localization of cFPGS mRNA and protein at cell protrusions. Using the MBSV6-loop reporter system and fluorescence microscopy, we demonstrate that following folate deprivation, cFPGS mRNA is retained in the endoplasmic reticulum, whereas upon 15 min of folate repletion, this mRNA is rapidly translocated to cell protrusions in a 3′UTR- and actin-dependent manner. The actin dependency of this folate-induced mRNA translocation is shown by treatment with Latrunculin B and inhibitors of the Ras homolog family member A (RhoA) pathway. Upon folate repletion, the FPGS 3′UTR GQs induce an amoeboid/mesenchymal hybrid cell phenotype during migration and invasion through a collagen gel matrix. Targeted disruption of the 3′UTR GQ motifs by introducing point mutations or masking them by antisense oligonucleotides abrogated cell protrusion targeting of cFPGS mRNA.

**Conclusions:**

Collectively, the GQ motifs within the 3′UTR of FPGS regulate its transcript and protein localization at cell protrusions in response to a folate cue, inducing cancer cell invasive phenotype. These novel findings suggest that the 3′UTR GQ motifs of FPGS constitute an attractive druggable target aimed at inhibition of cancer invasion and metastasis.

**Supplementary Information:**

The online version contains supplementary material available at 10.1186/s12915-023-01525-1.

## Background

Directional single-cell migration is an orchestrated process pivotal for development, establishment and maintenance of proper organization of multicellular organisms, immune response, and disease progression, including cancer cell migration and metastasis [1–4]. Among the major regulators of cell migration are members of the Rho family of small guanosine triphosphatases (GTPases), primarily Ras homolog family member A (RhoA), Ras-related C3 botulinum toxin substrate 1 (Rac1), and cell division control protein 42 homolog (CDC42) [5–7]. These GTPases undergo carboxymethylation which is crucial for cell migration [8, 9], affecting protein activation [9, 10], protein half-life [11], and subcellular protein localization [10, 12].

Protein methylation requires S-adenosylmethionine (SAM, AdoMet) as the methyl donor [13]; this one carbon methyl unit originates from 5-methyl-tetrahydrofolate (5-CH_3_-THF) [14, 15], which is the dominant circulating THF cofactor [16]. THF cofactors serve as one-carbon donors in a multitude of key biosynthetic processes such as the de novo biosynthesis of nucleotides and amino acids [14], one of which is L-methionine that is further converted into SAM.

Most eukaryotic cells are auxotrophic for folates [17]; thus, they must obtain folates from their diet through specified influx transport systems [18]. However, since folates can be rapidly exported out of cells, their intracellular retention absolutely relies on the unique enzyme folylpolyglutamate synthetase (FPGS) which is essential for their cellular homeostasis [19]. FPGS catalyzes the successive addition of glutamate residues to the ɣ-carboxyl group of folates, resulting in their polyglutamylation [20] and intracellular accumulation up to three orders of magnitude higher than in the blood [21, 22]. Hence, FPGS is vital for the proliferation of normal and malignant cells.

The FPGS gene is translated into two isoforms, differing by an N-terminal leader sequence which directs the enzyme to mitochondria [23, 24], where FPGS mediates the retention of necessary folates for glycine biosynthesis [24]. The shorter isoform, termed cytosolic FPGS (cFPGS), was considered as a soluble protein, pivotal for purine and pyrimidine nucleotide biosynthesis [25]. We have recently shown that cFPGS is associated with the cellular cytoskeletal network and intracellular membranes, allowing the regulated transport of cFPGS to locations of folate metabolism, including the sites of folate uptake at the plasma membrane, the nucleus, the outer mitochondrial membrane, and the ER [26]. While our previous study demonstrated microtubule-dependent transport of the cFPGS protein, we herein show the actin-dependent transport of the cFPGS mRNA to cell protrusions. This protrusion-localization of the cFPGS mRNA requires the presence of its 3′UTR, and in particular two G-quadruplex (GQ) motifs found therein.

In the latter respect, GQs are composed of 4 runs of three (GGG_4_) or four (GGGG_4_) adjacent guanines (G-tracts) which spontaneously self-organize into four-stranded DNA/RNA structures [27, 28]. Bioinformatic analyses suggested that 300,000 GQ motifs are present in the human genome [29]. Importantly, it has been shown that these GQ motifs are not randomly dispersed throughout the genome, but are rather densely distributed in many key regulatory regions [30], including replication origins [31], telomeres [32], promoters, as well as 5′ and 3′UTRs [33, 34]. It has been well established that GQs which are present in the 3′UTR of mRNAs can regulate the subcellular localization of these transcripts in certain cells like neurons [35, 36].

Low serum folate levels have been shown to be associated with increased tumorigenicity [37–41] and enhanced metastasis [41–43]. Invasiveness and metastasis may be induced in the search for folates via chemotactic mechanisms. In this respect, we herein uncovered that the 3′UTR of FPGS, the GQ motifs in particular, induce cell invasion in response to pulse folic acid (FA) repletion, suggesting a role in tumor progression, migration, and metastasis under low folate conditions in the tumor microenvironment. This suggests a role for FPGS, not only in intracellular folate retention and homeostasis, but also in folate sensing and chemotaxis, highlighting FPGS 3′UTR GQ motifs as an attractive druggable target for inhibition of cancer migration and invasion.

## Results and discussion

### G-quadruplex elements in the 3′UTR of FPGS mRNA are required for its translocation to cell protrusions

**Fig. 1.**
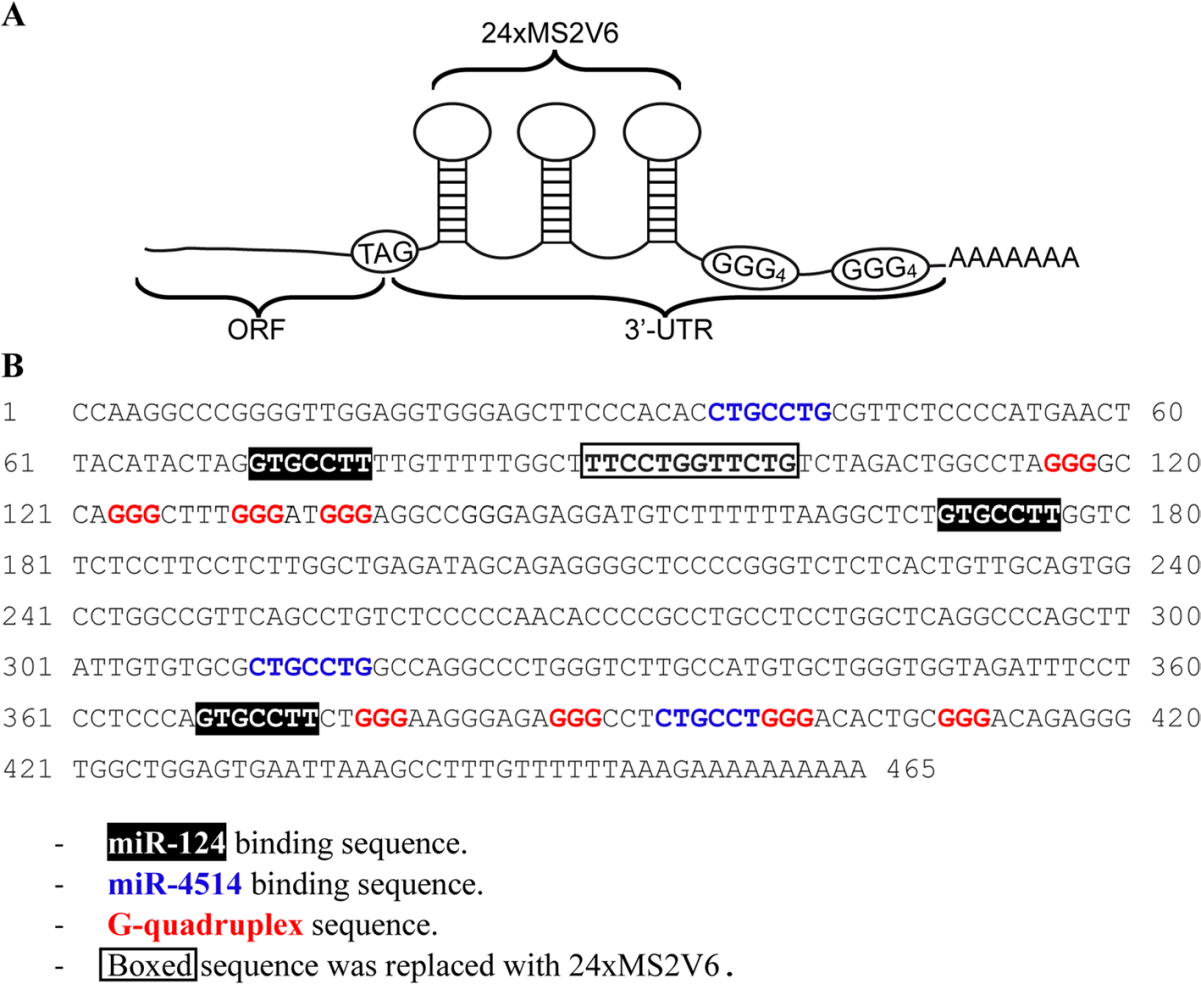
The 3′UTR of FPGS. **A** Schematic illustration of the 3′UTR and 24xMS2 sequences added to the FLAG-cFPGS expression vector. **B** Prediction of regulatory elements within the 3′UTR of FPGS and the insertion position of the 24xMS2 sequence

In our recent study [26], multiple actin binding/modulating proteins were identified by immunoprecipitation as cFPGS interactors, including cofilin 1 (CFL1), vinculin (VCL), fascin (FSCN1), cortactin (CTTN), IQ motif containing GTPase-activating protein 1 (IQGAP1), and talin-1 (TLN1). Actin was not identified as an interactor, although immunofluorescence (IF) microscopy did demonstrate the co-localization of cFPGS and actin on sub-membranous actin stretches. Since many of these proteins are components of the actin machinery that regulates the formation and stabilization of cell protrusions for migration (i.e., pseudopodia, filopodia) [5, 44] and invasion (i.e., invadopodia) [45, 46], we hypothesized that cFPGS might localize to said protrusions to sustain cell migration and invasion in response to specific cues. Given that various protrusion-localized proteins were shown to be locally translated at the cell edges following the transport of their mRNA [47], e.g., CFL1 [48], VCL [49], actin-related protein 2/3 complex (Arp2/3) [50], and β-actin [51], we set to explore whether this is also true for cFPGS. The primary element shown to govern cellular mRNA localization is the 3′UTR; this was shown extensively in neuronal cells [52–54] and for protrusion-localized proteins such as β-actin [55], CFL1 [48], RAB13 [56], and STAT3 [57, 58]. Hence, we utilized the well-established MBSV6-MCP reporter system [59] to determine the subcellular localization of cFPGS mRNA. To this end, the 3′UTR of FPGS was subcloned into the pRK5/FLAG-cFPGS (F-cFPGS) expression vector [26] and the sequence of 24xMS2V6 loops was inserted therein, as detailed in the “Methods” section (Fig. 1A) to yield the desired F-MS2 construct. The point of insertion of the 24xMS2V6 loops was selected carefully, to avoid the disruption of any regulatory element in the 3′UTR (Fig. 1B).

**Fig. 2.**
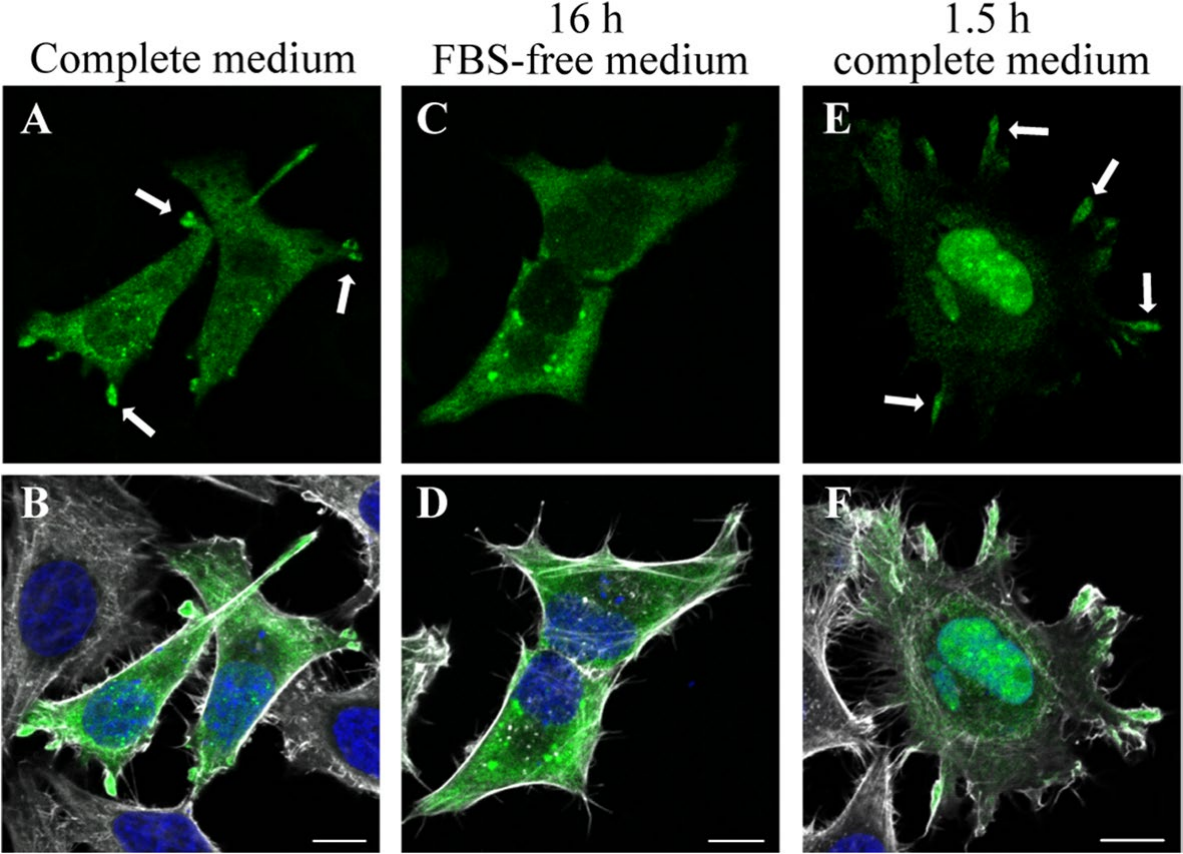
Confocal microscopy imaging of the subcellular localization of exogenous cFPGS mRNA harboring its native 3′UTR. HeLa cells co-transfected with FLAG-cFPGS-3′UTR-24xMS2 (F-MS2) and MCP-GFP were grown in full growth medium (**A**, **D**), serum-free medium (16 h, **B**, **E**), and serum-repleted medium (1.5 h, **C**, **F**). Fixed cells were stained with Hoechst 33342 (nuclei, blue fluorescence, 405 nm) and phalloidin (white, 630 nm) and were scanned using a confocal microscope (×63 magnitude). F-MS2 mRNA was detected by the green fluorescence of bound MCP-GFP (488 nm). Arrows point to cell protrusions with accumulated mRNA. Scale bars denote 10 μm. Shown are representative images of three independent experiments

To gain a qualitative proof of concept that cFPGS mRNA localizes at cell protrusions, we induced cell migration and pseudopodia formation by the routinely used method of serum starvation and repletion. F-MS2 and MS2-coat protein (MCP)-GFP co-transfected cells were grown over night in serum-free medium, after which they were incubated for 1.5 h in complete growth medium, fixed, and scanned using a confocal microscope. The subcellular localization of the exogenous cFPGS mRNA was monitored by following the green MCP-GFP fluorescence, and compared between cells that were grown under complete medium (Fig. 2A, B), serum-deprived (Fig. 2C, D), and serum-repleted (Fig. 2E, F) conditions. Actin staining was used to visualize the boundaries of the cells. In full growth medium, the F-MS2 mRNA was evenly distributed in the cells, with occasional high accumulation foci at cell protrusions (Fig. 2A, arrows). Whereas under serum deprivation, cells displayed less protrusions as previously shown [60], and F-MS2 mRNA was absent from the cell periphery (Fig. 2C). In contrast, upon serum repletion, F-MS2 mRNA was transported and accumulated at the edges of the newly formed, serum-induced filopodia (Fig. 2E, arrows). Consistently, FPGS mRNA was previously found to be enriched in protrusions of highly metastatic hepatocellular carcinoma cells [58].

Following the demonstration of the possible regulated transport of cFPGS mRNA to cell protrusions, we set to identify the element(s) within the 3′UTR of FPGS that govern(s) this translocation. Previous studies implicated GQ sequences in the 3′UTR of various neuronal genes, as the binding elements for RNA binding proteins (RBPs), that allowed the regulated transport of mRNAs to neuronal projections [35, 61]. It is reasonable that the same mechanism exists in non-neuronal cells, as several RBPs that were identified as GQ-binding proteins (GQBPs) [62–64] also transport RNA molecules to protrusions of migrating cells, such as fragile X mental retardation protein 1 (FMRP/FMR1) [57], or are a component of ribonucleoprotein complexes transported to cell protrusions, such as the RNA helicase DEAD box protein 3, X isoform (DDX3) [65], and the oncogene fused in sarcoma (FUS) [66]. Since the 3′UTR of FPGS is predicted to harbor two GQ sequences, we analyzed the 3′UTR of various protrusion-localized RNAs [48, 50, 56–58, 67–73] for the presence of GQ elements and compared their score to that of FPGS using the QGRS Mapper tool (Table 1). Although two-quartet GQs (i.e., GG_4_) have been recently identified and shown to form secondary structures [74–76], they have yet to be implicated in RNA transport and have low prediction scores; hence, we restricted the analyses to a minimum of three G runs (i.e., GGG_4_) and a maximum GQ length of 45 bp. Although the functional importance of the GQ elements shown in Table 1 has not been experimentally validated, except for the GQ of CAMK2A [35, 77], the mRNAs of CAMK2A, CFL1, and STAT3 are dependent on their 3′UTR for transport to cell protrusions [48, 57, 72], and all three are binding substrates of FMRP [57, 78, 79]; the latter has been shown to bind the majority of its RNA substrates through a GQ element [36, 61, 62, 80]. The actin crosslinkers filamin A and B (FLNA and FLNB) [81] were also found among FMRP’s binding substrates [82].

**Table 1:** G-quadruplex sequences found by QGRS Mapper in the 3′UTR of cell protrusion-localized RNAs

Gene	Accession	Position^**a**^	Length	QGs	G-score	Ref^**b**^
FPGS	NM_004957	116	22	**GGGGCCAGGGCTTTGGGATGGG**	70	58, current
		377	36	**GGGAAGGGAGAGGGCCTCTGCCTGGGACACTGCGGG**	70	
CFL1	NM_005507	89	29	**GGGCTGGGGGGATCCCAGCAGGGGGAGGG**	66	48, 58, 69
FSCN1	NM_003088	339	28	**GGGACGGTTGGGGGCTGGGAGCCCTGGG**	70	58, 70
		493	23	**GGGAAGGGAAGCTGTCGGGTGGG**	65	
		670	19	**GGGCGGGTAGGGGTGTGGG**	70	
FLNA	NM_001456	233	20	**GGGAGGGGTGAGGGATGGGG**	71	58, 69
FLNB	NM_001457	1028	44	**GGGTCTTTGGCCTGAAAGTTGGGAATGGTTGGGGGAGAGAAGGG**	62	58, 69
RAB13	NM_002870	57	40	**GGGAGTGAGCAGGGGAGAAATAGCAGAGGGGCTTGGAGGG**	67	56, 69, 71, 72
ACTR2	NM_005722	399	29	**GGGATGGGGGGTGGTTCGGGATGGGTGGG**	71	50, 70, 73
		564	33	**GGGTTTTGTTGCGGGGGGGAGGGTAACAATGGG**	68	
		1819	39	**GGGCCCGTACCTACTGGCAGTTGGGTTCAGGGAAATGGG**	57	
ARPC4	NM_005718	96	20	**GGGAGTTGGGTTGGGGTGGG**	70	69, 72
		626	44	**GGGGCCCAAAAGCCGGGCAACCTCTGGCTACAGGGGCCATTGGG**	63	
STAT3	NM_139276	248	28	**GGGGATGTGGCGGGGGGTGGCTAGAGGG**	64	57, 58
		1989	43	**GGGCTTACCATTGGGTTTAAATCATAGGGACCTAGGGCGAGGG**	70	
CAMK2A	NM_015981	335	37	**GGGCTGGTGCCCACCAGGGGCTGGGGAGAAGGAGGGG**	63	67, 68
		886	39	**GGGTCAGGTTGGAAGGGGTGTAGGAAGAGGGGTGAGGGG**	66	
		2674	39	**GGGTGGGCAGCCACCTGGTGCCACCACAGGGCACCAGGG**	52	
		3134	30	**GGGGGGGGCGGGTGGGATGGGAAGAAGGGG**	72	
CAPZB	NM_004930	453	26	**GGGGTTGGGGGTCGTGGGGACAGGGG**	107	58, 70, 72
		547	20	**GGGGTGGGGGCTGGGGGGGG**	106	

*FPGS* folylpolyglutamate synthase, *CFL1* cofilin 1, *FSCN1* fascin, *FLNA* filamin A, *FLNB* filamin B, *RAB13* Ras-related protein Rab-13, *ACTR2* actin-related protein 2, *ARPC4* actin-related protein 2/3 complex subunit 4, *STAT3* signal transducer and activator of transcription 3, *CAMK2A* calcium/calmodulin-dependent protein kinase type II subunit alpha, *CAPZB* F-actin-capping protein subunit beta

^a^Nucleotide position within the 3′UTR

^b^Reference papers demonstrating the protrusion localization/enrichment of each RNA

As evident from Table 1, the G-scores of the GQs of FPGS are comparable and even higher than those of CFL1, FLNA, FLNB, STAT3, and CAMK2A. Functional UTR GQs have been shown to be selectively constrained [33] and it has been suggested that universal selection for GQ formation in the UTRs of eukaryotic genomes may be related to the biological functions of GQs [83]. Indeed, an initial examination of the 3′UTR sequences of FPGS orthologs suggests that the GQ motifs are conserved around the same position in Mammalia, and even in Lepidosauria (Additional file 1: Table S1). In order to evaluate the conservation of the GQs in FPGS 3′UTR during vertebrate evolution, we obtained the 3′UTR sequences of FPGS homologs from diverse vertebrate species from the RefSeq database and analyzed them using QGRS mapper to predict the top two scoring GQs. To test whether the predicted GQs were a reflection of the G/C content of the 3′UTR or a specific preference for GQ-forming sequences, we further compared the real 3′UTR sequences to sequences with the same composition that were randomly shuffled. Comparison of the QGRS mapping scores has shown that the FPGS 3′UTR had a significantly strong preference to form two non-overlapping GQs than expected by chance in most amniote species (Additional file 2: Figure S1). In most cases, the top two scoring GQs also had a better score than that predicted by chance in the sequences with the same nucleotide composition. This shows that the sequence in amniotes evolved under conditions that preferentially retain the potential to form two distinct GQs, likely due to their functional roles in subcellular RNA localization. Collectively, these data suggest that the FPGS 3′UTR GQ sequences should be considered functional regulatory elements.

**Fig. 3.**
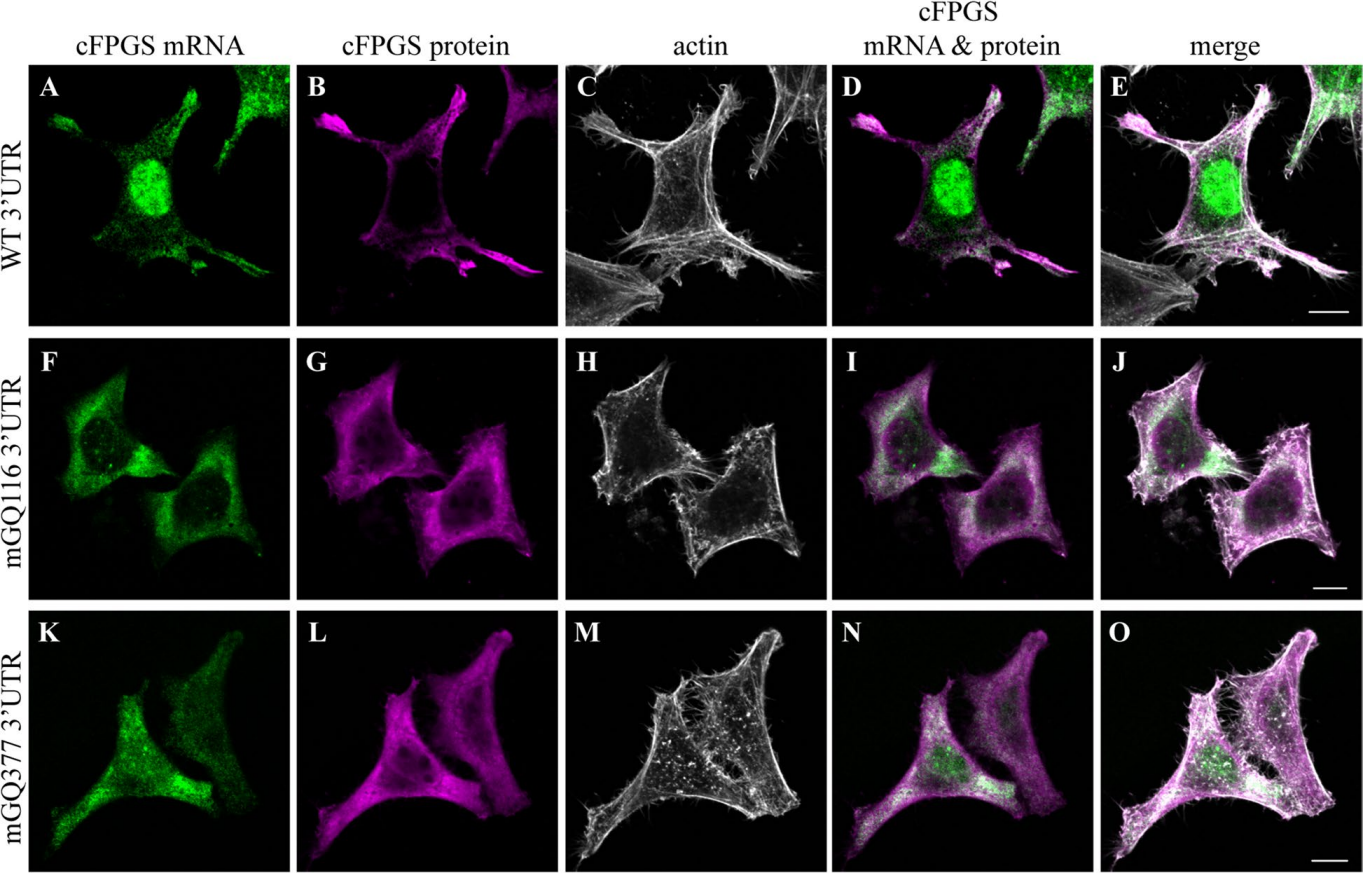
Disruption of the GQ elements at the 3′UTR of cFPGS mRNA abolishes its transport to cell protrusions. HeLa cells were co-transfected with MCP-GFP and FLAG-cFPGS-3′UTR-24xMS2 (F-MS2) harboring either the WT UTR (**A**–**E**), a mutated GQ at position 116 (mGQ116, **F**–**J**), or a mutated GQ at position 377 (mGQ377, **K**–**O**). Following 16 h of serum deprivation, cells were supplemented with complete growth medium for 1.5 h, fixed and subjected to IF microscopy. The localization of F-MS2 mRNA is indicated by MCP-GFP (green fluorescence, 488 nm), FLAG-cFPGS protein was detected by an anti-FLAG antibody (magenta, 405 nm), and F-actin was stained by DyLight 650 Phalloidin (white, 630 nm). Cells were scanned using a confocal microscope (×63 magnitude). The scale bars denote 10 μm. Shown are representative images of three independent experiments

To explore the possible contribution of these GQ elements to the transport of cFPGS mRNA to cell protrusions, we mutated each element separately on the F-MS2 expression vector and repeated the fetal bovine serum (FBS)-induction assay. Herein, we used IF microscopy to visualize the cell’s boundaries with filamentous actin (F-actin) along with the cFPGS protein. As depicted in Fig. 3, the disruption of each GQ was sufficient to abrogate the transport of FPGS mRNA to cell protrusions upon serum repletion. The mRNA appeared to be retained in the cell body, presumably in the ER area (Fig. 3 F and K); this coincides with our previous hypothesis, suggesting that cFPGS is translated and transported through the ER-Golgi network [26]. Each of the two GQ elements appears to have a pivotal role in the subcellular transport of FPGS mRNA. This implies either of the following: (1) a cooperative binding of the GQs by two RBPs [84], (2) the two GQs oligomerize to a higher order structure which is bound/stabilized by an RBP [85–87], or (3) binding of the two GQs by a single RBP harboring multiple binding sites, resulting in 3′UTR bending [88, 89]. To ensure complete abolishment of cFPGS mRNA transport, we generated a double GQ mutant (F-MS2-dmGQ) and used this construct upon subsequent experiments. Interestingly, the exogenous cFPGS protein (detected by an anti-FLAG antibody) colocalized with its cognate mRNA (Fig. 3 D, I, N), suggesting the localized translation of cFPGS, as was shown for various protrusion-localized proteins [68, 90].

### cFPGS mRNA transport to cell protrusions is induced upon FA repletion and is GQ- and F-actin-dependent

**Fig. 4.**
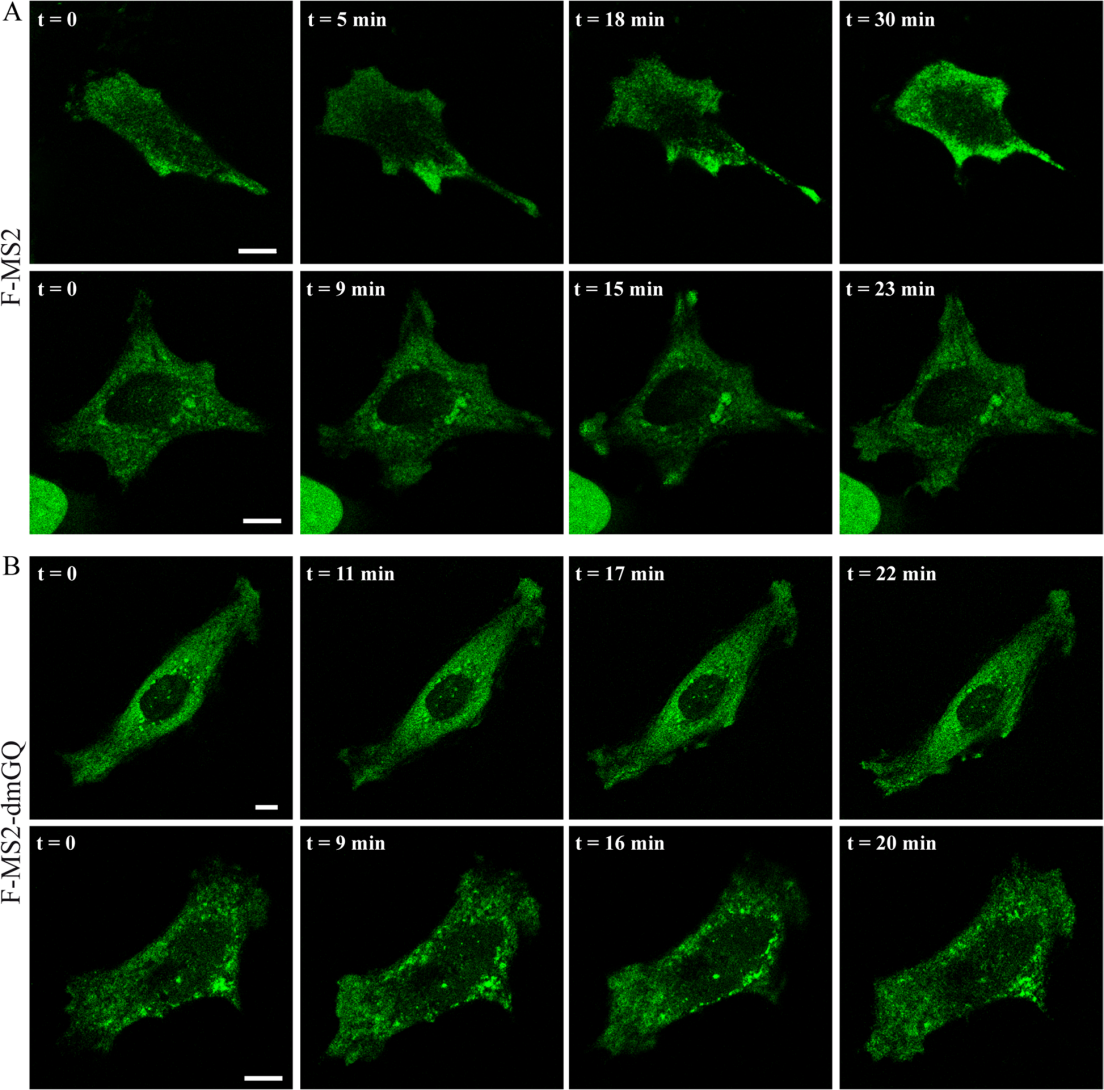
Time-lapse depiction of FA-induced cFPGS mRNA accumulation in a cell protrusion. HeLa cells deprived of FA for 13 days were co-transfected with MCP-GFP along with either F-MS2 (**A**) or F-MS2-dmGQ (**B**). The next day, the distribution of cFPGS mRNA (MCP-GFP, 488 nm) was monitored in single cells by live fluorescence imaging before (*t* = 0) and after the addition of 2μM FA to the growth medium. Cells were scanned with a confocal microscope (×63 magnitude). The scale bars denote 10 μm

FPGS is a key regulator of intracellular folate retention and homeostasis [19] and FA is a known chemoattractant [91–94] crucial for the migration and invasion of A549 non-small cell lung cells [10] as well as prostate cancer cells [95]. Moreover, a 24-h FA deprivation in HeLa cells consistently resulted in an increase in both migration and invasion [96]. Based on these findings, we explored the subcellular localization of cFPGS mRNA upon FA repletion. To this end, HeLa cells were deprived of FA for 14 days; on day 13, cells were transfected with F-MS2 and studied the next day by live fluorescence microscopy imaging before and after the addition of 2 μM FA. Figure 4A depicts examples of individual cells, at different time points following FA addition, where cFPGS mRNA accumulation coincided with the elongation of a cell protrusion (Fig. 4A, top) or occurred in preexisting protrusions (Fig. 4A bottom). RNA accumulation at cell protrusions was most apparent around ~15min, after which the accumulated mRNA became dispersed. The experiment was repeated with the use of the F-MS2-dmGQ construct (Fig. 4B), which resulted in the lack of cFPGS mRNA accumulation during the same time frame. We have therefore chosen to conduct further experiments using a 15-min FA repletion pulse.

**Fig. 5.**
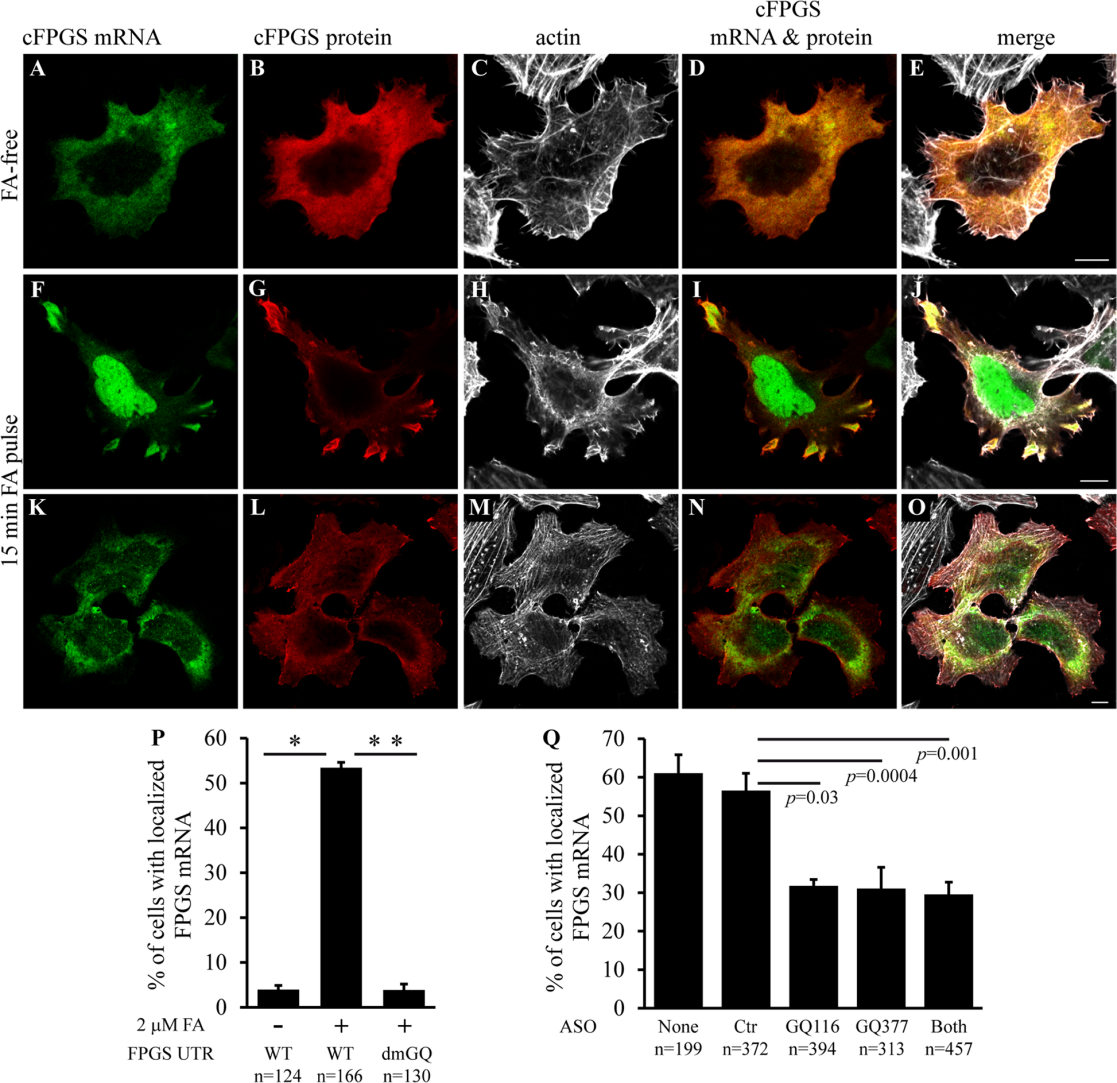
The 3′UTR GQ elements are required for FA-induced cFPGS mRNA transport to cell protrusions. **A–P** On day 12 of FA deprivation, HeLa cells were co-transfected with MCP-GFP and F-MS2 (**A**–**J**) or F-MS2-dmGQ (**K**–**O**). Each transfection was divided into 2 wells and left to recover. At day 14, control cells remained FA-free (**A**–**E**) and the rest were subjected to a 15-min FA pulse (**F**–**O**), prior to fixation and IF microscopy. **Q** On day 13 of FA-deprivation, HeLa cells were co-transfected with MCP-GFP and F-MS2 along with ASOs as indicated. The next day, cells were subjected to a 15-min FA pulse, prior to fixation and microscopy. The localization of cFPGS mRNA was indicated by MCP-GFP (green, 488 nm), FLAG-cFPGS protein was detected by an anti-FLAG antibody (red, 543 nm), and F-actin was stained by DyLight 650 Phalloidin (white, 630 nm). Cells were scanned and manually counted using a confocal microscope (×63 magnitude). The scale bars denote 10 μm. **P**, **Q** Percentage of cells displaying protrusion-localized cFPGS mRNA. Shown are the mean percentage of cells ± S.D. from three independent experiments, *n* = total number of cells counted. *p <0.0006, **p < 0.00003

Next, to be able to efficiently quantify the FA-dependent transport of cFPGS mRNA to cell protrusions, and to evaluate the requirement of the 3′UTR GQ elements to this transport, we performed IF microscopy. HeLa cells deprived of FA were co-transfected with MCP-GFP along with either F-MS2 or F-MS2-dmGQ and subjected to a FA repletion pulse, following which cells were fixed and reacted with an anti-FLAG antibody. The results obtained with the FA pulse (Fig. 5) were identical to those after FBS repletion (Fig. 3). Fifty-three percent of cells harboring F-MS2 displayed protrusion-localized cFPGS mRNA following a FA pulse (Fig. 5F, P), compared to only 3.8% of F-MS2-dmGQ harboring cells (Fig. 5K, P), the same percentage as in FA-starved cells (i.e., 3.9%, Fig. 5A, P). To further corroborate these findings, we utilized antisense oligonucleotides (ASOs) against the FPGS 3′UTR GQs. ASOs have been recently utilized for sequence-specific inhibition of RNA GQ folding, thus reducing protein translation [97–99]. We here employed the same technique with the aim to abrogate the FA-induced cell protrusion localization of cFPGS mRNA. While the non-targeted ASO-Ctr had no effect on cFPGS mRNA localization following a 15-min FA-pulse repletion, each of the GQ-targeted ASOs alone reduced the percentage of cells with protrusion-localized mRNA by ~50% (Fig. 5Q). No further reduction was observed when both ASOs were used together, corroborating their cooperative role in cFPGS mRNA localization in response to FA repletion. Interestingly, upon FA repletion cells harboring the F-MS2-dmGQ construct (Figs. 3, 4, and 5) presented less protrusions per cell than cells harboring the F-MS2 construct. When comparing the two populations of cells (50 cells from each group), the average number of protrusions for the WT UTR was 7 ± 1.7 vs. 2.7 ± 1.2 for the dmGQ UTR (*p* = 1.9 × 10^−26^), suggesting a role for cFPGS mRNA in protrusion formation.

**Fig. 6.**
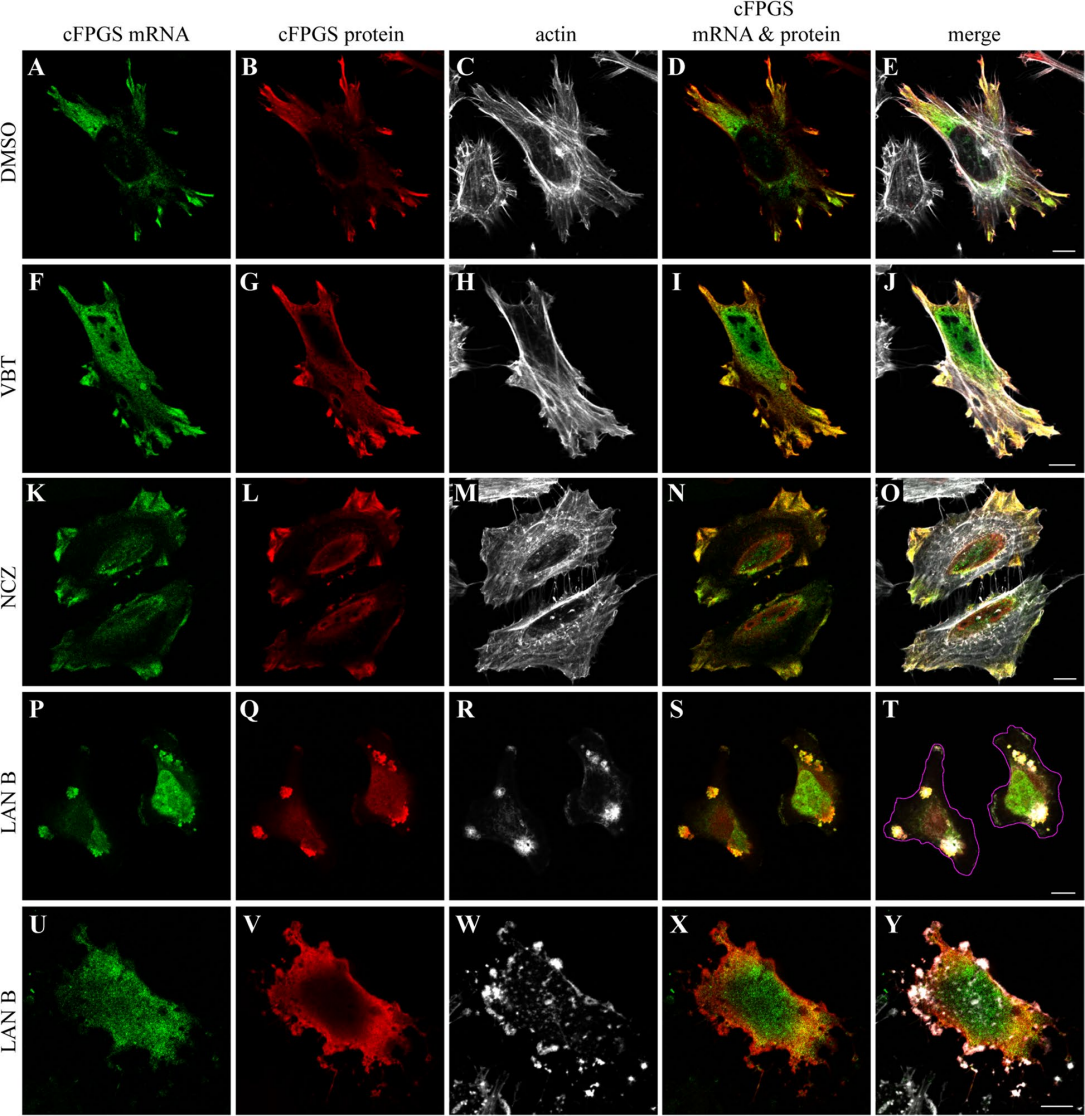
cFPGS mRNA transport to cell protrusions is dependent on actin filaments. HeLa cells, deprived of FA for 12 days, were co-transfected with MCP-GFP along with either F-MS2 (**A**–**T**) or F-MS2-dmGQ (**U**–**Y**). The next day, each cell group was split into different wells and left to grow in FA-free medium for an additional 24 h. Cells were pre-treated for 1 h with 0.1% DMSO (vehicle, **A**–**E**), 40 μM VBT (**F**–**J**), 10 μM NCZ (**K**–**O**), or 250 nM LAN B (**P**–**Y**) and supplemented with 2 μM FA for 15 min before fixation and IF microscopy. The localization of cFPGS mRNA is indicated by MCP-GFP (green fluorescence, 488 nm), FLAG-cFPGS protein was detected by an anti-FLAG antibody (red, 405 nm), and F-actin was stained by DyLight 650 Phalloidin (white, 630 nm). **T** The silhouette of the cells was outlined with a purple line. Cells were scanned using a confocal microscope (×63 magnitude). The scale bars denote 10 μm. Shown are representative images of three independent experiments

Out of the three most studied RBPs mediating the transport of mRNA to cell protrusions, i.e., FMRP [61, 79, 100]; adenomatous polyposis coli, APC [101–103]; and Zipcode binding protein 1, ZBP1 [104, 105], only FMRP recognizes and binds RNA GQs [61, 62, 106]. Since FMRP is dependent on microtubules for mRNA transport [107, 108], and as we have previously demonstrated the association of the cFPGS protein with the cellular cytoskeletal network, primarily with microtubules [26], we explored the dependence on microtubules for the FA-induced protrusion localization of cFPGS mRNA (Fig. 6). Following FA deprivation, transfected cells were pre-treated with the microtubule depolymerizing agent vinblastine (VBT) [109] for 1 h before FA repletion; cells were then fixed and subjected to IF microscopy. Surprisingly, VBT did not hinder the accumulation of cFPGS mRNA and protein in cell protrusions (Fig. 6F–J). In fact, the percentage of cells with localized mRNA was greater with the microtubule depolymerizing agent than in control cells (66.5% vs. 53.4% *p* = 0.02, *n* = 140 and 166 cells for Ctr and VBT, respectively). To verify that the microtubules were disrupted by VBT, cells were reacted with an anti-α-tubulin (α-Tub) antibody (Additional file 2: Figure S2). Indeed, following VBT treatment, microtubules appeared as short rod-like crystals as previously shown (Additional file 2: compare Figure S2L & Q to S2B & G) [110, 111], while cFPGS protein accumulated in cell protrusions (Additional file 2: Figure S2K & P). The microtubule-independent transport of cFPGS mRNA was also confirmed with the microtubule dynamics disrupting agent nocodazole (NCZ) [112] (Fig. 6K–O).

The disruption of microtubules not only increased the percentage of cells with protrusion-localized cFPGS mRNA after a 15-min FA pulse, but also increased the residence time of said mRNA in protrusions. While drug-free cells exhibited mRNA dispersion after ~30 min (Fig. 3), with no cells harboring localized mRNA after 2 h (Additional file 2: Figure S3 A, C), VBT-treated cells retained the protrusion and the localized mRNA even after 3 h (Additional file 2: Figure S3, E, G). Previous studies demonstrated that filopodia remain intact and motile after disruption of microtubule with VBT and/or NCZ [113, 114]. Although many studies describe the transport of mRNA to cell protrusions, we could not find any publication describing the fate of mRNA after its local translation; does it undergo dispersion or degradation? Our current results suggest that while microtubules are not required for cFPGS mRNA transport to cell protrusions, they are required for mRNA retrograde transport/degradation.

As ZBP1 was shown to use actin filaments for RNA transport [51, 105], in addition to microtubule, we pre-treated the cells with the actin polymerization inhibitor latrunculin B (LAN B) before FA-repletion (Fig. 6P–T). Under these inhibitory conditions, both cFPGS mRNA and protein co-localized with actin in large aggregates without reaching the cell edges. Since mutating the GQ elements in the FPGS 3′UTR disrupted mRNA transport to cell protrusions (Fig. 5), we hypothesized that it will abolish the physical connection between the mutated transcript and the actin cytoskeleton. Indeed, when cells were transfected with the MS2-dmGQ construct prior to LAN B treatment, cFPGS mRNA was distributed within the cells and not aggregated along with actin (Fig. 6U–Y). Moreover, the cFPGS protein, translated from the MS2-dmGQ construct, did not aggregate with actin as did the protein from the WT transcript (Fig. 6, compare V to Q), suggesting that cFPGS is not only locally translated at cell protrusions, as shown for many mRNAs [68], but is also translated while in transit, as was previously demonstrated [90].

As detailed in the “Methods” section, a point mutation resulting in a V525A substitution (V567 in the mitochondrial FPGS isoform) was introduced close to the C-terminus of cFPGS, to facilitate the subcloning of the 3′UTR into the pRK5 expression vector. When we performed IF microscopy with this V525A mutated construct, termed F-cFPGS-V525A, to ensure that the mutation did not interfere with the localization of the cFPGS protein, we were surprised to find it in perinuclear vesicles (Additional file 2: Figure S4, A-D). This vesicular localization of cFPGS-V525A resembled that of the WT cFPGS which we previously reported, following treatment with LAN B and brefeldin A [26]. Based on our hypothesis that cFPGS translation and sorting occur through the ER-Golgi network [26], the V525A substitution might disrupt the ER export signal of cFPGS, as a single C-terminal valine was shown to operate as a strong ER export signal [115]. Consistently, previous findings revealed that a C-terminal valine is involved in the rapid and selective ER export of the FXYD7 protein, a regulator of Na^+^/K^+^ ATPase [116]. Indeed, cFPGS V525 which is located in a conserved hydrophobic patch, is completely conserved across eukaryotes, suggesting it is an essential residue. This substitution was corrected before further experiments were performed; however, since we had indications that cFPGS mRNA undergoes translation while in transit and/or protrusion-localized translation, the FLAG-cFPGS-V525A-3′UTR expression vector was used to explore this possibility. FA-depleted cells were transfected with said plasmid and used for IF microscopy before and after a pulse repletion with FA (Additional file 2: Figure S4). Under FA starvation, the cFPGS-V525A protein appeared in intracellular clusters (Additional file 2: Figure S4, E and K) which were juxtaposed to the ER marker calnexin (CANX) (Additional file 2: Figure S4, G, H and M). However, following a 15-min FA pulse, cFPGS-V525A assumed a dispersed localization and reached the cell periphery (Additional file 2: Figure S4, P and U, arrows). This suggests that following FA repletion, cFPGS-V525A was not retained in the ER since it was not translated therein. These results support both the ER as the first station for cFPGS mRNA and the regulation of its site of translation—either in the ER or cell protrusions.

### FA-induced cFPGS mRNA transport to cell protrusions is dependent on the RhoA pathway

Actin-dependent RNA transport to cell protrusions is not very common and has been primarily studied with β-actin mRNA [117, 118], which is transported by the actin-dependent RBP ZBP1 [51, 105]. The dependence on actin for cFPGS mRNA transport is interesting, as the Krupenko group showed that the actin cytoskeleton is affected by dietary folates through the phosphorylation state of CFL1, i.e., folate deprivation results in decreased levels of inactive phosphorylated CFL1 (pCFL1) [10, 119]. Activated CFL1 depolymerizes and severs actin filaments, whereas pCFL1 dissociates from G/F actin as a result of reduced affinity [120]. The regulation of CFL1 phosphorylation is governed by a RhoA-dependent cascade [10, 121], whereas the localization and activation of RhoA is regulated by its methylation state [10, 122]; inactivated RhoA is anchored to the ER membrane via a prenyl moiety and is released upon prenylcysteine carboxymethylation [10]. FA, as a one-carbon unit donor in multiple cellular reactions, is required for SAM-dependent methylation [14, 123], and as such folate deprivation results in an ER-bound inactive RhoA [10]. The dependency of RhoA on carboxymethylation led Oleinik et. al. to conclude that RhoA is an immediate cellular sensor of FA status [10].

**Fig. 7.**
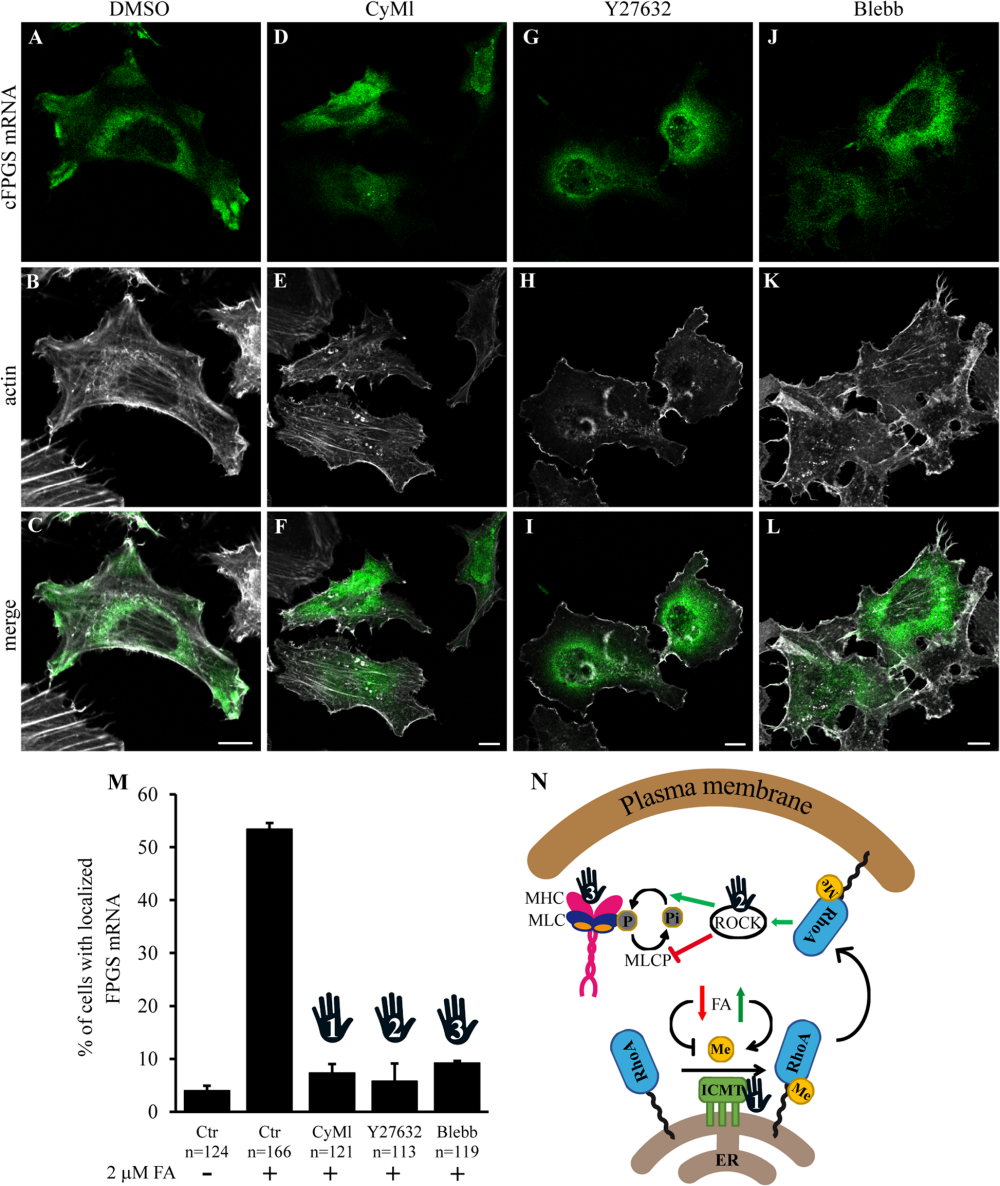
The RhoA-ROCK-Myosin II pathway is required for the transport of cFPGS mRNA to cell protrusions. HeLa cells, deprived of FA for 12 days, were co-transfected with MCP-GFP and F-MS2. The next day, transfected cells were split into 5 wells and left to grow in FA-free medium for an additional 24 h. Cells were pre-treated for 1 h with 0.1% DMSO (vehicle, **A**–**C**), 30 μM CyMl (**D**–**F**), 20 μM Y27632 (**G**–**I**), or 15 μM Blebb (**J**–**L**) and supplemented with 2 μM FA for 15 min before fixation and microscopy. The localization of cFPGS mRNA is indicated by MCP-GFP (green fluorescence, 488 nm) and F-actin is stained by DyLight 650 Phalloidin (white, 630 nm). Cells were scanned using a confocal microscope (×63 magnitude). The scale bars denote 10 μm. Shown are representative images of three independent experiments. **M** Confocal microscopy was used to count cells with protrusion-localized cFPGS mRNA. Shown are the mean percentage of cells ± S.D. from three independent experiments, *n* = total number of cells counted. All *p* values ≤ 0.001. Stop signs denote pharmacological inhibition detailed in **N**. **N** Illustration of the RhoA-ROCK-Myosin II pathway and the sites of pharmacological inhibition used (marked by a stop sign). RhoA is anchored to the ER membrane via a prenyl moiety and is retained therein under folate deprivation. Elevated levels of tetrahydrofolate cofactors increase the availability of S-adenosylmethionine (Me) leading to the carboxymethylation of RhoA by ICMT, which can be inhibited by CyMl. Following carboxymethylation RhoA is released from the ER and translocates to the plasma membrane, where it activates ROCK1/2; the latter can be inhibited by Y27632. ROCK activates myosin II by both phosphorylating the myosin light chain (MLC) and inhibiting the activity of MLC-phosphatase (MLCP). Myosin ATPase activity can be inhibited by Blebb

As β-actin mRNA delivery to cell protrusions was shown to be dependent on Rho-associated protein kinase (ROCK) and myosin II [117, 118], and since RhoA activity can be regulated by FA, it is plausible that RhoA senses the folate cue which initiates the transport of FPGS mRNA to the periphery. We utilized several inhibitors of the RhoA pathway to determine whether it is required for the transport of cFPGS mRNA to cell protrusions (Fig. 7A): Cysmethynil (CyMl) is a potent inhibitor of protein-S-isoprenylcysteine O-methyltransferase (ICMT) [124, 125] which carboxymethylates RhoA [122, 126] among other proteins. Y27632 is a *bona fide* inhibitor of the RhoA effectors ROCK 1/2 [127]. Blebbistatin (Blebb) is an inhibitor of the ATPase and gliding motility activities of myosin II [128, 129]. Treatment with any of these three inhibitors resulted in a significant reduction in the percentage of cells exhibiting cFPGS mRNA localization at cell protrusions upon FA repletion (Fig. 7B), indicating the importance of the RhoA pathway to FPGS mRNA transport to cell protrusions.

We propose a feedback loop between RhoA and cFPGS: Under FA deprivation, cFPGS is retained at the ER—from which it can be transported, in a microtubule-dependent rout, to intracellular locations of folate metabolism—where the FA sensor RhoA resides. Upon FA repletion and methylation of RhoA, it activates the pathway leading to cFPGS mRNA transport and translation at cell peripheries, where the FPGS enzyme—by rapid polyglutamylation—enables the accumulation of folates necessary for actin remodeling and cell migration.

### The 3′UTR GQs of FPGS are required for a FA-induced cell invasion phenotype

**Fig. 8.**
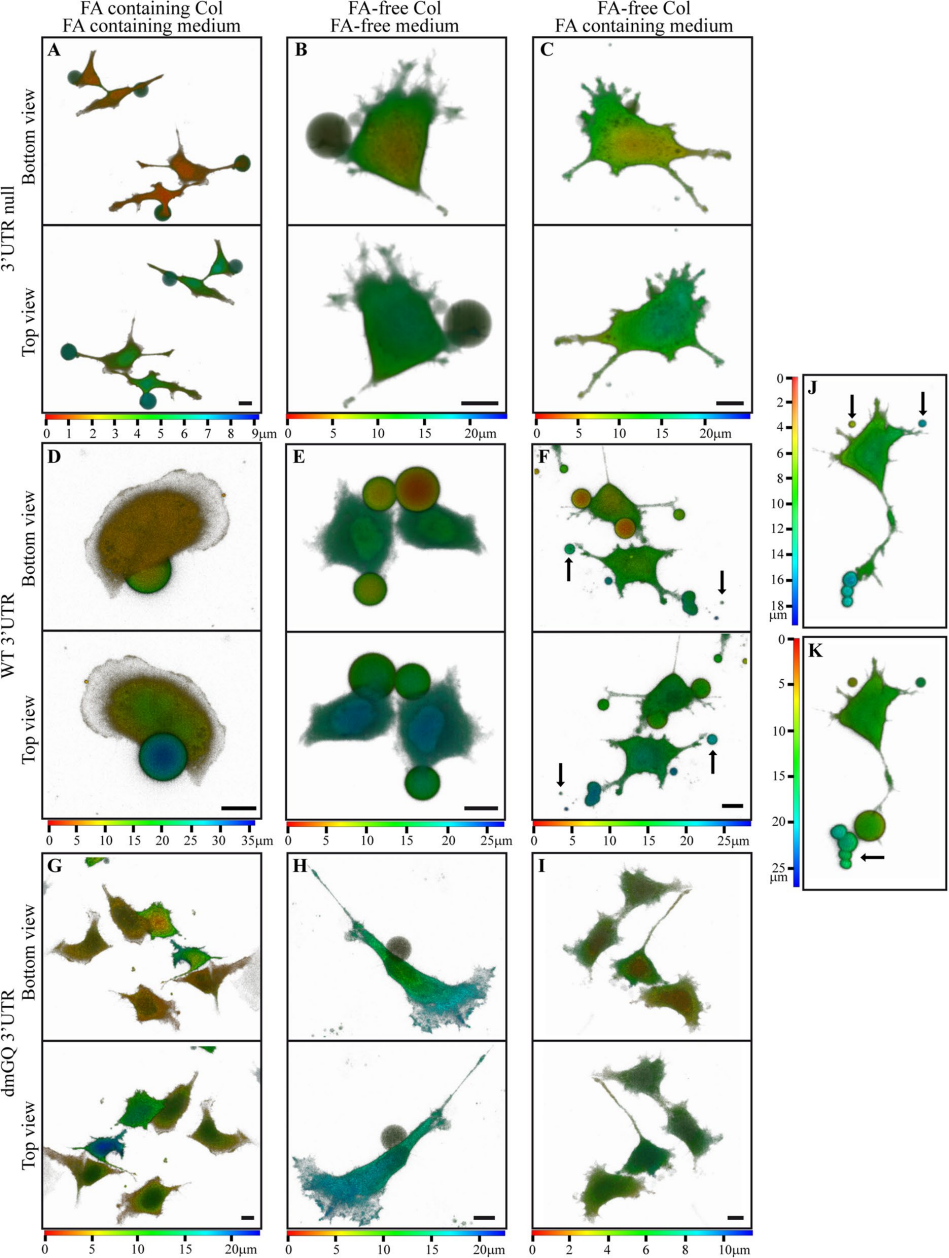
The 3′UTR of FPGS enhances cell migration under confined conditions. FA-deprived HeLa cells, co-transfected with either F-cFPGS (3′UTR-null) and BFP (**A**–**C**), F-MS2 and GFP (**D**–**F**, **J**, **K**), or F-MS2-dmGQ and RFP (**G**–**I**), were overlaid with FA-containing collagen and medium (**A**, **D**, **G**), FA-free collagen and medium (**B**, **E**, **H**), or FA-free collagen and FA-containing medium (**C**, **F**, **I**–**K**) as detailed in Figure S1. Following 40 h of incubation, cells were visualized by confocal microscopy using focus stacking (i.e., Z-stacks), and 3D semi-translucent images were generated. Each image is shown from both a bottom and top view, with a color gradient representing the depth within the image. **J**, **K** Images of the same cell with a 2.5-h interval, during which a string of vesicles detached from the cell protrusion, and a new bleb formed at the tip of the protrusion. Arrows point at vesicles released from cell protrusions. The scale bars denote 10 μm. Transition through the Z-stacks can be seen in the supplementary movies S1-S9

To examine the validity of this putative feedback loop and explore the possible FA-dependent induction of an invasive phenotype, we utilized an inverted invasion assay [130, 131]. The following are the considerations for using this specific assay in which cells plated at the bottom of the well invade the top collagen (Col) matrix: (1) The widely used Boyden chamber Transwell invasion assay utilizes a commercially available basement membrane matrix, such as Matrigel or Cultrex, which consist of DMEM medium [132, 133]. DMEM contains ~9 μM FA, hence curtailing any assessment of FA-induced invasion potential. In contrast, the inverted invasion assay we utilized employs pure Col solubilized in acetic acid solution; this allows for the exogenous addition of a FA-containing medium on top of the FA-free collagen. (2) The basis for refraining from using the conventional Transwell system is the poor diffusion of medium components including FA from the bottom chamber with FA-containing medium into the upper Col matrix, precluding a FA-driven chemotactic effect and hence preventing any possibility of migration and invasion towards the Col matrix. The current inverted assay allows for the gravitational percolation of the growth medium into the Col gel sieve. As such, the bottom-plated cells can undergo an upwardly directed migration and invasion into the FA-containing Col in a chemotactic manner, as compared to a FA-free collagen. In traditional invasion assays, the Matrigel is supplemented with 1% FBS, thus allowing the cells to invade the growth factor-containing matrix and migrate close to the Transwell membrane where a micro-gradient of growth hormones induces the directional invasion of the cells to the underlaid growth medium containing 10% FBS. (3) The inverted invasion assay allows physical confinement where cell migration is dependent on the RhoA pathway [134, 135]. A concentration of 2.4 mg/ml type I collagen, used in this protocol, should generate a confined space with ~3-μm diameter pores [136–138]. Various cell lines, including HeLa, switch to a fast amoeboid migration phenotype, which includes the formation of a stable bleb [135, 139, 140]. FA-deprived cells were transfected separately with a construct for either cFPGS without a 3′UTR (F-cFPGS), F-MS2, or F-MS2-dmGQ. Each construct was co-transfected with an expression vector harboring a fluorescent protein to allow monitoring by fluorescence microscopy, i.e., F-cFPGS & BFP, F-MS2 & GFP, and F-MS2-dmGQ & RFP. Cells were mixed, seeded on glass plates, and overlaid with Col under three conditions: FA-containing Col immersed in FA-containing medium, FA-free Col immersed in FA-free medium, and FA-free Col immersed in FA-containing medium (Additional file 2: Figure S5). Cells were incubated for 40 h before they were visualized by live confocal imaging using focus stacking to record images at different focal planes (i.e., Z-stacks), allowing the generation of 3D images, and measure the spreading distance of each cell (Fig. 8 and Additional files 3, 4, 5, 6, 7, 8, 9, 10, 11: movies S1-S9).

When FA was present in the cells’ immediate surroundings (i.e., within the Col), cells harboring F-cFPGS and F-MS2 developed a single bleb per cell, either from the cell body or from a protrusion, and the blebs developed in an upward direction, i.e., the leading edge (Fig. 8A, D, Additional file 3, 6: movies S1, S4). This is consistent with leader bleb-based migration [139, 141, 142], although the cells here were not round and maintained normal cell protrusions. Metastatic cancer cells were previously shown to have the ability to switch between protrusion types to optimize their migration in different environments [143, 144] and even exhibit an amoeboid/mesenchymal hybrid phenotype [145] under the control of RhoA [146]. Indeed, for cancer metastasis to occur, malignant cells must traverse a range of tissue environments with diverse physicochemical properties; this is accomplished, at least in part, by cells adjusting their migration mode to one that is best suited to the actual environment [147]. Blebs were shown to act as sites of extracellular matrix adhesion and re-organization during hybrid mode cell invasion [145]. In contrast, cells expressing F-MS2-dmGQ failed to develop any bleb (Fig. 8G, Additional file 9: movie S7). Each cell spanned ≤10 μm on the *Z* axis, excluding F-MS2-harboring cells which had very large blebs. Clearly, in the presence of FA, there was no need for the cells to spread out as FA was readily available. Under FA deprivation, the blebs were under the cells, following and not leading (Fig. 8B, E, H, Additional files 4, 7, 10: movies S2, S5, S8). This was the only condition under which some F-MS2-dmGQ harboring cells developed blebs (Fig. 8, compare H to G and I); however, they were relatively small. Interestingly, while the blebs that were formed under the expression of F-MS2 seemed to have higher content density than the cell body (i.e., higher fluorescent intensity, Fig. 8E, Additional file 7: movie S5), the blebs that were formed in the other cells displayed very weak fluorescence, making them appear almost transparent (Fig. 8B, H, Additional files 4, 10: movies S2, S8), suggesting a different composition of these blebs resulting from uneven distribution of the cytosolic content between the cell body and the bleb. Interestingly, leading pseudopods of invading cells are characterized by a higher cell mass density [148]. In the pursuit of FA, cells spread a longer distance reaching ~20 μm, and yet the blebs were close to the cell body. The most significant changes in cellular behavior elicited by the three FPGS constructs were observed when FA was supplemented in the growth medium, slowly diffusing into the Col matrix as a vitamin chemoattractant. Cells expressing the UTR-null cFPGS transcript resembled those growing in the presence of FA, i.e., one upward-directed leader bleb per cell and short distance spreading (Fig. 8C, Additional file 5: movie S3). When the 3′UTR GQs were mutated, cells did not develop any blebs and cell spreading was < 10 μm (Fig. 8I, Additional file 11: Movie S9). In stark contrast, cells expressing FPGS with the WT UTR exhibited multiple blebs, with varying sizes, locations, and directions (Fig. 8F, Additional file 8: movie S6), with each cell spreading ≥15 μm. Additional file 2: Figure S6 allows to compare the morphology of cells harboring the WT UTR and dmGQ-UTR within the same growth area when FA-containing medium was added on top of the Col gel. FPGS WT-UTR, but not the dmGQ-UTR, induced the formation of leading blebs and invasion higher into the Col matrix. F-MS2 bearing cells developed protrusions from which blebs were formed and occasionally released (Fig. 8F, J, K, arrows); a string of vesicles formed at the tip of a cell protrusion (Fig. 8J) was released when a new bleb formed at said protrusion (Fig. 8K). Tumor cell blebbing and vesicle shedding are known phenomena that facilitate migration and invasion [149–151], specifically the very large oncosomes which contain matrix metalloproteinases, among others [152, 153].

A leader bleb can force its way forward by a net transfer of cytoplasm in the direction of migration [144], hence the leading edge (pseudopods and blebs) was shown to have a higher cell mass density [148]. We therefor determined the fluorescence intensity of the cell body and the protruding blebs of the Col-invading cells as an indication of the cell mass (Table 2). Cells transfected with cFPGS expression vectors harboring either no-3′UTR or mutated 3′UTR exhibited lower fluorescence in the blebs compared to the cell body, with 3′UTR-dmGQ displaying a 4-fold lower bleb fluorescence. In contrast, cells harboring the WT-3′UTR exhibited up to 4-fold higher bleb fluorescence when compared to the cell body, corroborating the requirement of the FPGS 3′UTR for the development of invasive leading blebs.

**Table 2:** Fluorescence intensity ratios between the protruding blebs and cell body of cells in 3D Col gel

Growth condition	FPGS 3′UTR	Fluorescence intensity ratio^**a**^ bleb/cell body	***p*** value vs. WT 3′UTR
FA-containing Col and medium	Null	0.75 ± 0.22	4.60E−12
	WT	3.91 ± 0.58	
	dmGQ	N.A.	
FA-free Col and medium	Null	0.58 ± 0.09	4.60E−08
	WT	4.03 ± 0.18	
	dmGQ	0.25 ± 0.08	2.50E−08
FA-free Col and FA-containing medium	Null	0.29 ± 0.08	2.20E−07
	WT	3.25 ± 0.58	
	dmGQ	N.A.	

*N.A.* not applicable since no blebs were visual

^a^The fluorescence intensity ratios presented are the mean values obtained from analysis of ~10 cells ± S.D.

## Conclusions

Our novel findings reveal that the GQ motifs in the 3′UTR of FPGS regulate its transcript and protein localization at cell protrusions in response to a folate cue, thereby inducing cancer cell invasion. Previous studies by the group of S. Mili have shown that cancer cell invasion requires RNA localization at cell protrusions and the invasive front [56, 102, 154], hence suggesting RNA localization as a target for interference with cancer cell invasion. Along this vein, the FPGS 3′UTR GQs emerge in our present study as an attractive druggable target for the design of novel therapeutics. While inhibiting FPGS activity may be harmful to healthy tissues, impairing the cell protrusion localization of its mRNA by ASOs, targeted to the GQ motifs may inhibit invasion and metastasis. In recent years, DNA and RNA GQs have gained much interest as targets for antiviral [155, 156] and anticancer therapy [157, 158]; studies include, among others, inhibition of the translation of COVID-19 nucleocapsid phosphoprotein [159], inhibition of the translation of vascular endothelial growth factor [160], and suppression of c-myc transcription [161]. Thus, disruption of the subcellular localization of well-defined mRNAs may emerge as a novel targeted anticancer treatment strategy which may be enhanced when combined with chemotherapeutic as well as immunotherapeutic agents.

## Methods

### Chemicals and materials

RPMI-1640 medium (#21875034) and fetal bovine serum (#10270106) were from Gibco, Life Technologies, Grand Isle, NY. Glutamine, penicillin G, and streptomycin sulfate were from Biological Industries, Beit-Haemek, Israel. FA-free medium (#R1145), dialyzed fetal bovine serum (#F0392), and VBT (# V1377) were from Sigma-Aldrich, St. Louis, MO, USA. NCZ (#sc-3518), LAN B (#sc-203318), Y27632 (#sc-281642), and CyMl (#sc-500804) were from Santa Cruz Biotechnology, Dallas, TX, USA. Blebb (#13013) was from Cayman Chemical, Ann Arbor, MI, USA. FA (#J62937) was from Alfa Aesar, Tewksbury, MA, USA.

### Tissue culture

Human cervical carcinoma HeLa cells (American Tissue Culture Collection, Manassas, VA) were maintained in RPMI-1640 medium supplemented with 10% FBS, 2 mM glutamine, 100 units/ml penicillin G, and 100 μg/ml streptomycin sulfate in a humidified atmosphere of 5% CO_2_ at 37 °C. For folate deprivation, cells were grown in FA-free medium supplemented with 10% dialyzed FBS, 2 mM glutamine, 100 units/ml penicillin G, and 100 μg/ml streptomycin sulfate for 14 days.

### Online prediction tools and databases

The 3′UTR sequence of FPGS was analyzed for possible regulatory elements using the following online prediction tools: (1) The microRNA database *miRBase* was used to search for microRNA binding sites; URL: http://www.mirbase.org/ [162]. (2) The web-based *QGRS Mapper* program was used to predict and score RNA G-quadruplex elements; URL: https://bioinformatics.ramapo.edu/QGRS/index.php [163].

### Expression vectors

Primers used in this section are detailed in Table 3 (Sigma-Aldrich). Restriction enzymes were from New England Biolabs (Ipswich, MA, USA). Vectors and inserts were purified using the Wizard PCR & Gel cleanup kit (#A9281, Promega, Madison, WI, USA). Ligations were performed using the DNA Ligation kit 2.1 (# 6022, TaKaRa Bio, Shiga, Japan). All plasmids were sequenced by an ABI 3730xl DNA analyzer (Applied Biosystems, Waltham, MA, USA).

**Table 3:** Primers used in the current study

Application	Name	Sequence 5′-3′
3′-RACE	polyT adapter	GACGCGTGTGGACAGTCGATTTTTTTTTTTTTTTTTT
	FPGS EX11-up^a^	CAAAGGCATCCAGGCCAGG
	adapter	GACGCGTGTGGACAGTCGA
	FPGS Ex15-up	TCAGCCAAGGCCGAGACC
Cloning	NheI-UTR	TAAGCTAGCCACTGGCAGCCTGCACC
	UTR-HindII	AAAAAGCTTGACGCGTGTGGACAGTCG
	MS2-Fw	ACCATGATTACGCCAAGCTTG
	MS2-Rv	GTGAATTCGAGCTCGGTACCTC
	overhang-BFP	CAAACAACAAGATGTGGAAGGCGGTAG
	BFP-XhoI	CAAACTCGAGAATTAAGCTTGTGCCCC
SDM^b^	NheI-sdm	GCCATCCATGTGCTAG***C***CACTGGCAGCCTGCACCTG
	NheI-fix	GCCATCCATGTGCTAG***T***CACTGGCAGCCTGCACCTG
	EcoRV-sdm	GGTGCCTTTTGTTTTTGG***A***T***A***TCCTGGTTCTGTCTAGACTGGCC
	GQ116-sdm	GATCTAGACTGGCCTAGG***TT***CCA***TT***GCTTTG***T***GATG***T***GAGGCCGGGAGAGGATGTC
	GQ377-sdm	CCTCCCAGTGCCTTCTG***T***GAAG***T***GAGAG***T***GCCTCTGCCTG***T***GACACTGCGGGACAGAG

^a^Ref [164]

^b^Only the sense sequence is given. SDM was performed with two complementary primers

Underlined sequences denote restriction sites

Nucleotides mutated by SDM are shown in bold and are italicized

The pRK5/FLAG-cFPGS expression vector (F-cFPGS), harboring the ORF of cFPGS with an N-terminal FLAG-tag, was previously described [26]. The pRK5/FLAG-cFPGS-3′UTR-MS2V6 vector (F-MS2), harboring the ORF of cFPGS with its 3′UTR and the MBSV6-loop system [59, 165] was generated as follows:

- Cloning of the 3′UTR sequence of FPGS was performed by 3′ rapid amplification of cDNA ends (3′-RACE) as follows: Following the isolation of total RNA from HeLa cells, using the TRI Reagent RNA Isolation Reagent (#T9424, Sigma-Aldrich), we performed reverse transcription with the High-Capacity cDNA Reverse Transcription Kit (#AB-4368814, Thermo Fisher Scientific, Waltham, MA, USA) primed by a polyT adapter primer with a customized extension instead of random primers. A first-round PCR was performed using HY TAQ READT MIX (# EZ-3007, hylabs, Rehovot, Israel) with a specific target forward primer (FPGS EX11-up) and an adapter primer. A second PCR was performed using the product from the first round as template, the adapter primer, and a second target primer (FPGS Ex15-up )[164], located downstream of the first. The final PCR products were resolved on a 1% agarose gel, purified, and cloned using the pGEM-T-easy ligation kit (# A1360, Promega).
- The 3′UTR sequence was subcloned into the F-cFPGS expression vector as follows: The UTR sequence was PCR-amplified from the pGEM vector using primers NheI-UTR & UTR-HindIII. For cloning purposes, we introduced a nucleotide change c.T2514C (NM_004957.6) introducing a NheI restriction site in the F-cFPGS vector (F-cFPGS-V525A), using the QuickChange II site-directed mutagenesis (SDM) kit (#200523, Agilent technologies, Santa Clara, CA, USA) and the NheI-sdm primers. Following a NheI-HindIII digestion of the vector and 3′UTR-insert and their ligation, an F-cFPGS-V525A-3′UTR plasmid was generated. We corrected the nucleotide change by SDM (NheI-fix primers) to generate the vector pRK5/FLAG-cFPGS-3′UTR.
- The subcloning of the 24xMS2V6 loops system was performed by PCR-amplifying the ~1.7 Kb MS2 sequence from the pET259-pUC57 24xMS2V6 plasmid (Addgene plasmid #104391) [59], using Q5 high-fidelity polymerase (#M0491, New England Biolabs) and the primers MS2-Fw & MS2-Rv, followed by digestion with SmaI & XbaI. An EcoRV restriction site was generated in the 3′UTR sequence of pRK5/FLAG-cFPGS-3′UTR by SDM with the EcoRV-sdm primers, followed by digestion with EcoRV & XbaI. Ligation yielded the vector F-MS2.

The F-MS2-mGQ116 and F-MS2-mGQ377 vectors, harboring mutated GQ sequences at the 3′UTR, were generated by SDM using primers GQ116-sdm and GQ377-sdm, respectively. Consecutive SDM reactions were used to generate the double mutant F-MS2-dmGQ. Mutating the GQ sequences did not affect the predicted miRNA-binding sites.

The pUbC-nls-ha-stdMCP-stdGFP (MCP-GFP) expression vector, harboring a synonymized tandem dimer MCP fused to synonymized tandem dimer GFP, was a gift from Prof. Robert Singer (Addgene plasmid # 98916) [166].

pcDNA3.1/ZNT1-Ruby was previously described [167]. pTurboRFP-C (#FP231) and pTurboGFP-N (#FP512) were from Evrogen (Moscow, Russia). To generate a pTurboBFP-C construct, BFP was PCR-amplified from mTagBFP2-TOMM20-N-10 (Addgene plasmid #55328) [168] using the Q5 high-fidelity polymerase and the primers hang-BFP & BFP-XhoI. The pTurboRFP-C vector and BFP insert were digested by AgeI and XhoI, and then ligated.

### Transfections

All transfections were carried out using linear polyethylenimine (PEI, MW 25,000) transfection reagent (#23966, Polysciences, Warrington, PA, USA). Cells were seeded in 24-well plates (4×10^4^ cells/well) 24 h prior to transfection. Transfections were performed using 1 μg DNA at a PEI-DNA ratio of 3:1. F-MS2 and MCP-GFP plasmids were co-transfected at a ratio of 2:1.

### Immunofluorescence (IF) and live imaging microscopy

Cells grown in complete growth medium or at day 12 of FA deprivation were transfected with the indicated expression vectors. After 16 h, the growth medium was replaced and cells were allowed to recuperate for 2 h, following which cells were trypsinized, and seeded in Eppendorf’s Cell Imaging 24-well plates (#EP0030741021, Hamburg, Germany). For FBS-deprivation, the growth medium was replaced with FBS-free medium for a 16-h incubation period. The next day (i.e., day 14 of FA deprivation), cells were either fixed (as detailed below) or imaged after/during FBS or FA repletion using a confocal Zeiss LSM 710 inverted microscope (×63 magnification, Oberkochen, Germany) during incubation at 37°C in an atmosphere of 5% CO_2_.

For IF microscopy, cells were washed with PBS and fixed for 15 min with a freshly prepared 4% formaldehyde solution. This was preceded by 1 h of pre-incubation with 0.1% DMSO (vehicle) or the following inhibitors: 40 μM VBT, 10 μM NCZ, 250 nM LAN B, 30 μM CyMl, 20 μM Y27632 or 15 μM Blebb, and 15-min co-incubation with 2 μM FA. Following fixation, cells were washed twice with PBS for 5 min, permeabilized with 0.1% Triton X-100 in PBS for 10 min followed by two washes with PBS. Cells were blocked for 1 h at room temperature (RT) with TBS buffer (10 mM Tris, 150 mM NaCl, pH 7.4) containing 20% skimmed milk and then incubated with primary antibodies for 1 h at RT: 1:330 mouse anti-FLAG M2 (#F1804, Sigma-Aldrich), 1:500 rabbit anti-α-tubulin (#ab4074, Abcam, Cambridge, UK), and 1:200 rabbit anti-calnexin (#ADI-SPA-860, Enzo Life Sciences, Farmingdale, NY, USA). Following three 5-min washes with PBS, cells were co-incubated with fluorescent secondary antibodies along with DyLight 650 Phalloidin (#12956, Cell Signaling Technology, Danvers, MA, USA), for 1 h at RT in the dark: 1:400 goat anti-mouse DyLight 405 #115-475-062, 1:400 donkey anti-mouse Cy3 #715-165-150, 1:400 donkey anti-rabbit Alexa Fluor 488 #711-545-152, or 1:400 donkey anti-rabbit DyLight 405 #711-475-152 (Jackson ImmunoResearch laboratories, West Grove, PA, USA). Cells were then washed three times with PBS for 5 min and once with DDW, topped with Fluoromount-G (#00-4958-02, Invitrogen, Carlsbad, CA, USA), and covered with 13-mm glass coverslips. Fluorescence was recorded using a confocal Zeiss LSM 710 microscope (×63 magnification), with excitation wavelengths of 405, 488, 543, and 630 nm. Focus stacking was performed with 1.5-μM intervals from the lowest plane in an upwards direction. Confocal microscopy images and movies were processed using the ZEN 3.0 SR black edition software (Carl Zeiss Vision GmbH, Oberkochen, Germany).

### GQ conservation analysis

For analysis of GQ conservation, we obtained the 3′UTR sequences of FPGS from different species from the Entrez RefSeq database. For each sequence, we used the command line version of the QGRS mapper (https://bioinformatics.ramapo.edu/QGRS/index.php) with default parameters to find the highest scoring GQ in the 3′UTR sequences as well as 100 randomly shuffled sequences of each 3′UTR. The two top-scoring GQs in the actual sequence were compared with those of the randomized sequences, computing an empirical *P* value for both the top-scoring G quadruplex, as well as for the sum of the scores of the two top-scoring GQs. If no GQ was found by QGRS mapper, a score of zero was assigned. Empirical *P* values were computed by comparing the score of the actual 3′UTR sequence with the randomized ones. Box plots show the median, 1st and 3rd quartiles, and whiskers extend to the further points in the 1.5 times the interquartile range.

### cFPGS mRNA localization analyses

To determine the percentage of cells with localized cFPGS mRNA, only cells with cytoplasmic MCP were considered; cells exhibiting only nuclear MCP were dismissed. Cells were scored as protrusion localized if the MCP signal was significantly higher in cell protrusion (one or more) than in the cell body.

### GQ targeting with antisense oligonucleotides

HeLa cells at day 13 of FA-deprivation were transfected with F-MS2 and MCP-GFP along with one of the following ASOs at a DNA ratio of 2:1:1, respectively: a non-targeted control ASO-Ctr which served as a negative control, 5′-ATTCCGGAATTGACTGACTGACTGA. ASO-GQ116, 5′-CTCTCCCGGCCTCCCATCCCAAAGC. ASO-GQ377, 5′-GTCCCGCAGTGTCCCAGGCAGAGGC (Integrated DNA Technologies, Coralville, IA, USA). Cells were also transfected with a combination of ASO-GQ116 and ASO-GQ377 or with F-MS2 and MCP-GFP alone, to verify that the control oligo had no impact on RNA localization. Six h after transfections, the growth medium was replaced with fresh FA-free medium. A 15-min FA pulse was given to the cells 24 h after transfection, following which they were fixed with formaldehyde as described above. Cells were then scanned using a confocal microscope and manually counted for cFPGS mRNA localization analysis.

### Inverted invasion assay

Inverted invasion assays were conducted according to the published protocol by McArdle et. al. [131]. Specifically, on day 10 of FA deprivation, cells were seeded in 24-well plates. The next day cells were co-transfected with either of the following expression vectors: F-cFPGS & pTurbo-BFP, F-MS2 & pTurbo-GFP, or F-MS2-dmGQ & pTurbo-RFP. On day 12, the growth medium containing the transfection reagent was replaced and the cells were allowed to recover for 2 h before they were trypsinized, mixed together, and seeded in three 35-mm glass-bottom plates (#200350, SPL Life Sciences, Gyeonggi-do, Korea). After 4 h, when the cells attached to the plate, the growth medium was removed and cells were overlaid with 150 μl Col hydrogel as follows: Using micropipette tips stored at −20^o^C, we combined on ice RatCol rat tail Type I collagen solution (#5153, Advanced BioMatrix, San Diego, CA, USA) at a final concentration of 2.4 mg/ml, one 10th volume of FA-free 10xRPMI-1640 medium, 30 mM NaHCO_3_ and FBS-containing RPMI-1640 medium either containing (one plate) or lacking FA (two plates); see Additional file 2: Figure S5 for the assay design. After 30 min of incubation at 37 °C, when the hydrogels solidified, a thin layer of grease was applied on the rim of each gel to prevent its detachment during the assay. Gels were immersed in growth medium either containing or lacking FA and the plates were incubated for 40 h in a humidified atmosphere of 5% CO_2_ at 37°C.

The first attempts included a formaldehyde fixation step; however, even 1 h long fixation did not affect the gel-invading cells, as was seen by the unsuccessful actin staining with phalloidin. Hence, we abandoned fixation and visualized the cells by live fluorescence imaging using an LSM 710 confocal microscope.

### Content density

Cells from the inverted invasion assays were analyzed for content density represented by the fluorescence of the exogenous fluorescent protein they express, i.e., BFP, GFP, and RFP. For the calculation of fluorescence intensity, the cell body was defined as the perinuclear region with the highest fluorescence. If multiple blebs were present per cell, the bleb with the highest fluorescence was selected for the analysis. The regions of interest, i.e., cell body and blebs, were marked manually and measured throughout the recorded Z-stacks using the ImageJ software (version 1.52i, Wayne Rasband National Institute of Health, Washington, DC, USA). The fluorescence value is the mean fluorescence recorded in each 3D area.

### Statistical analyses

For the quantification of changes in cell percentage with localized cFPGS mRNA, a two-tailed paired Student *t*-test was performed. For the number of protrusions per cell and cell content density, a two-tailed two-sample equal variance Student t-test was performed. *p* values ≤ 0.05 were considered statistically significant and are given in the Figure legends and Table 2.

## Supplementary Information

**Additional file 1: Table S1.** G-quadruplex sequences found by QGRS Mapper in the 3'UTR of FPGS orthologs.

**Additional file 2: Figures S1-S6. Figure S1.** G scores for GQ motifs in the 3’UTR of FPGS orthologues. **Figure S2.** IF microscopy of VBT treated cells. **Figure S3.** Live imaging of FPGS RNA localization in VBT treated cells. **Figure S4.** IF microscopy suggesting the localization of FPGS translation. **Figure S5.** Design of cell migration assay under collagen-confined conditions. **Figure S6.** Cell morphology within collagen gel. Legends to movies S1-S9.

**Additional file 3: Movie S1.** F-cFPGS & BFP harboring cells in FA-containing collagen.

**Additional file 4: Movie S2.** An F-cFPGS & BFP harboring cell in FA-free collagen.

**Additional file 5: Movie S3.** An F-cFPGS & BFP harboring cell in FA-free collagen immersed in FA-containing medium.

**Additional file 6: Movie S4.** An F-MS2 & GFP harboring cell in FA-containing collagen.

**Additional file 7: Movie S5.** F-MS2 & GFP harboring cells in FA-free collagen.

**Additional file 8: Movie S6.** F-MS2 & GFP harboring cells in FA-free collagen immersed in FA-containing medium.

**Additional file 9: Movie S7.** F-MS2-dmGQ & RFP harboring cells in FA-containing collagen.

**Additional file 10: Movie S8.** An F-MS2-dmGQ & RFP harboring cell in FA-free collagen.

**Additional file 11: Movie S9.** F-MS2-dmGQ & RFP harboring cells in FA-free collagen immersed in FA-containing medium.

## Data Availability

All data generated or analyzed during this study are included in this published article (and its supplementary information files).

## Abbreviations

GTPases: Guanosine triphosphatases
RhoA: Ras homolog family member A
Rac1: Ras-related C3 botulinum toxin substrate 1
CDC42: Cell division control protein 42 homolog
SAM/AdoMet: S-Adenosylmethionine
5-CH3-THF: 5-Methyl-tetrahydrofolate
FPGS: Folylpolyglutamate synthetase
cFPGS: Cytosolic FPGS
GQ: G-quadruplex
FA: Folic acid
CFL1: Cofilin 1
VCL: Vinculin
FSCN1: Fascin
CTTN: Cortactin
IQGAP1: IQ motif containing GTPase-activating protein 1
TLN1: Talin-1
IF: Immunofluorescence
ARP2/3: Actin-related protein 2/3 complex
RAB13: Ras-related protein Rab-13
STAT3: Signal transducer and activator of transcription 3
F-cFPGS: pRK5/FLAG-cFPGS
F-MS2: pRK5/FLAG-cFPGS-3′UTR-MS2V6
MCP: MS2-coat protein
RBP: RNA binding protein
GQBP: GQ-binding protein
FMRP/FMR1: Fragile X mental retardation protein 1
DDX3: DEAD box protein 3, X isoform
FUS: Fused in sarcoma
CAMK2A: Calcium/calmodulin-dependent protein kinase type II subunit alpha
FLNA: Filamin A
FLNB: Filamin B
ACTR2: Actin-related protein 2
ARPC4: Actin-related protein 2/3 complex subunit 4
CAPZB: F-Actin-capping protein subunit beta
FBS: Fetal bovine serum
F-actin: Filamentous actin
F-MS2-dmGQ: Double GQ mutant F-MS2
ASO: Antisense oligonucleotide
APC: Adenomatous polyposis coli
ZBP1: Zipcode binding protein 1
VBT: Vinblastine
α-Tub: α-Tubulin
NCZ: Nocodazole
LAN B: Latrunculin B
CANX: Calnexin
pCFL1: Phosphorylated CFL1
ROCK: Rho-associated protein kinase
CyMl: Cysmethynil
ICMT: Protein-S-isoprenylcysteine O-methyltransferase
Blebb: Blebbistatin
Col: Collagen
