# Supplementary material for: Folylpolyglutamate synthetase mRNA G-quadruplexes regulate its cell protrusion localization and enhance a cancer cell invasive phenotype upon folate repletion
Source: BMC Biol. 2023 Feb 1;21:13. doi: 10.1186/s12915-023-01525-1 (PMC9889130; doi:10.1186/s12915-023-01525-1)
Supplement: Supplementary file 2 — Additional file 2: Figures S1-S6. Figure S1. G scores for GQ motifs in the 3’UTR of FPGS orthologues. Figure S2. IF microscopy of VBT treated cells. Figure S3. Live imaging of FPGS RNA localization in VBT treated cells. Figure S4. IF microscopy suggesting the localization of FPGS translation. Figure S5. Design of cell migration assay under collagen-confined conditions. Figure S6. Cell morphology within collagen gel. Legends to movies S1-S9. [file 12915_2023_1525_MOESM2_ESM.pdf]

BMC Biology, Additional file 2

Folylpolyglutamate synthetase mRNA G-quadruplexes regulate its cell protrusion localization and enhance a cancer cell invasive phenotype upon folate repletion

Michal Stark[1], May Levin[1], Igor Ulitsky[2] and Yehuda G. Assaraf[1*]

This file includes:

Supplementary Figures and Legends S1-S6.

Legends for Supplementary Movies S1-S9.

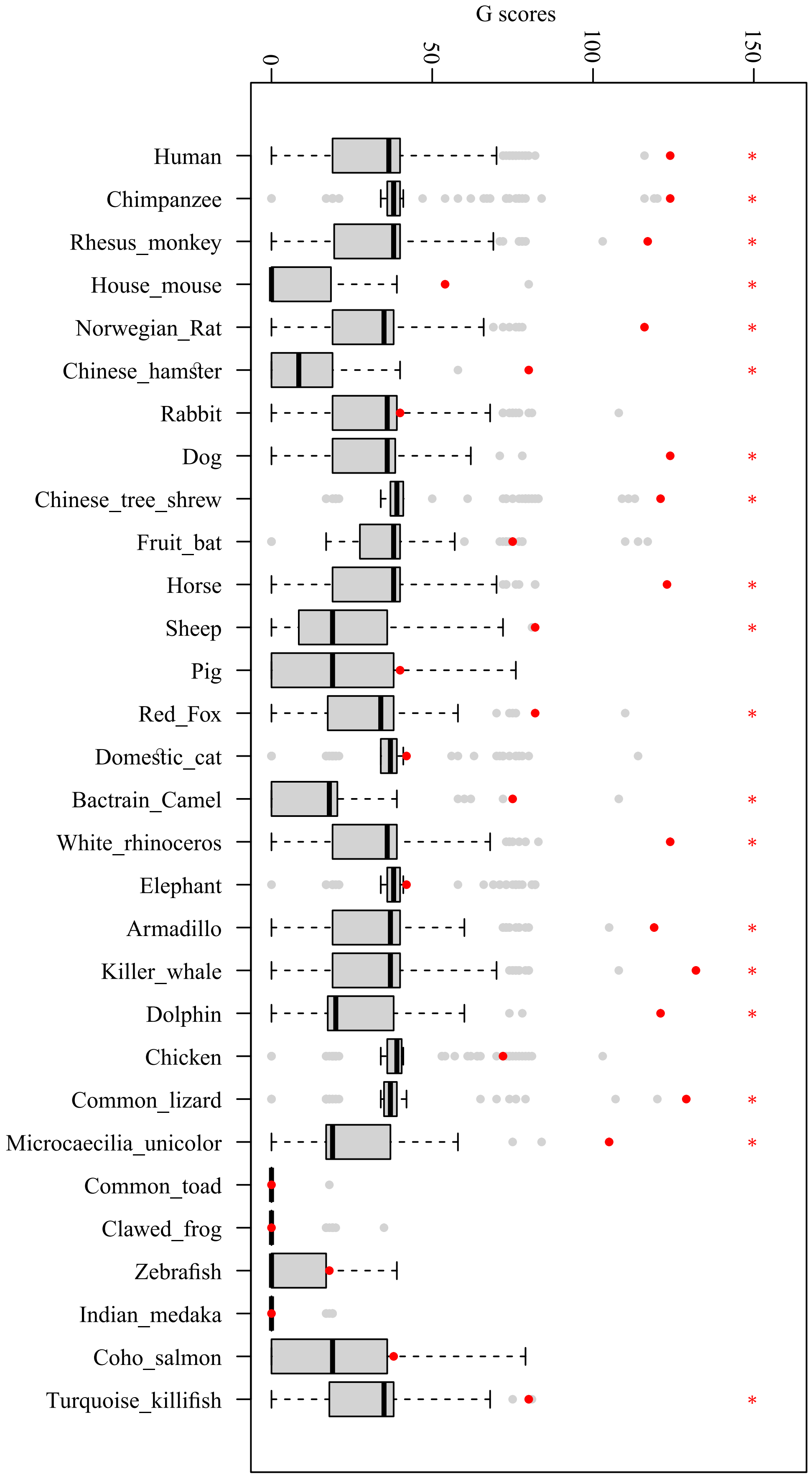

Figure S1: G scores for GQ motifs in the 3'UTR of FPGS orthologues. Shown are the sums of the top two highest scoring non-overlapping GQs in FPGS 3'UTR sequences from the indicated species (red dots) and in 100 randomly shuffled sequences (box plots and gray dot outliers), as predicted by QGRS Mapper. Box plots show the median, 1st and 3rd quartiles, and whiskers extend to the further points in the 1.5-times the interquartile range. Species where empirical P-values were <0.05 are marked by red asterisks.

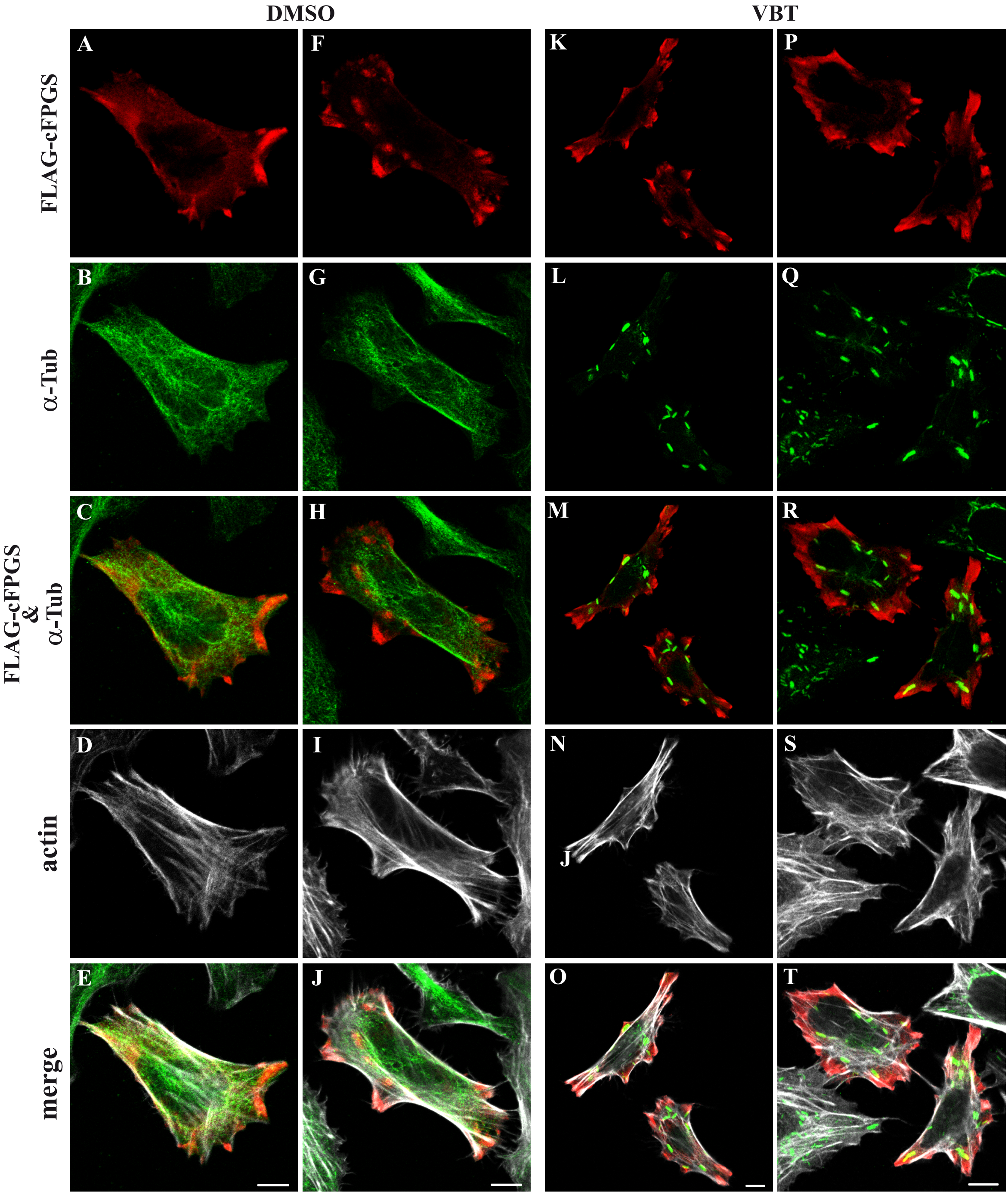

Figure S2: FA-induced cFPGS transport to cell protrusions is independent of microtubule filaments. HeLa cells, transfected with F-MS2 were deprived of FA for 14 days. Cells were pre-treated for 1 h with 0.1% DMSO (A-J) or 40 µM VBT (K-T) and supplemented with 2 µM FA for 15 min before fixation and IF microscopy. FLAG-cFPGS protein was detected by an anti-FLAG antibody (red, 405 nm), microtubules were detected by an α-Tub antibody (green, 488 nm) and F-actin was stained by DyLight 650 Phalloidin (white, 630 nm). Cells were scanned using a confocal microscope (x63 magnitude). The scale bars denote 10 µm.

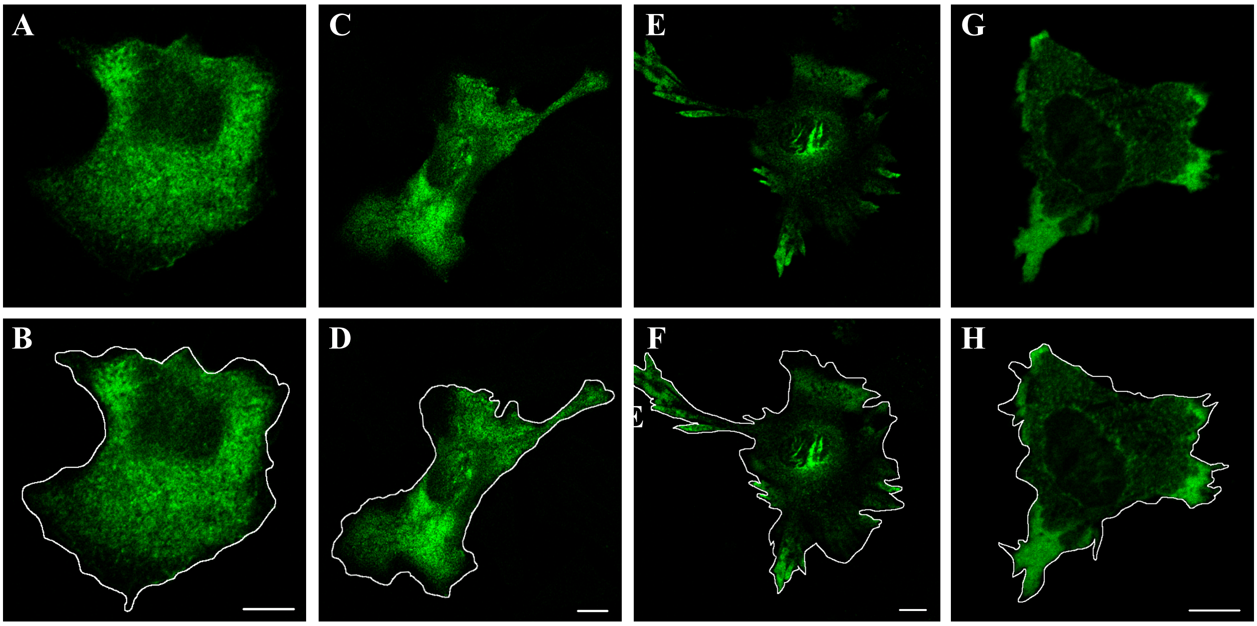

Figure S3: cFPGS mRNA is retained in cell protrusions for longer periods upon VBT treatment. HeLa cells, co-transfected with F-MS2 and MCP-GFP were deprived of FA for 14 days. Cells were pre-treated for 1 h with 0.1% DMSO (A-D) or 40 μM VBT (E-H) and supplemented with 2 μM FA for 2 h (A-D) or 3 h (E-H). Cells were scanned using a confocal microscope (x63 magnitude). The scale bars denote 10 μm. The silhouette of the cells is outlined (bottom).

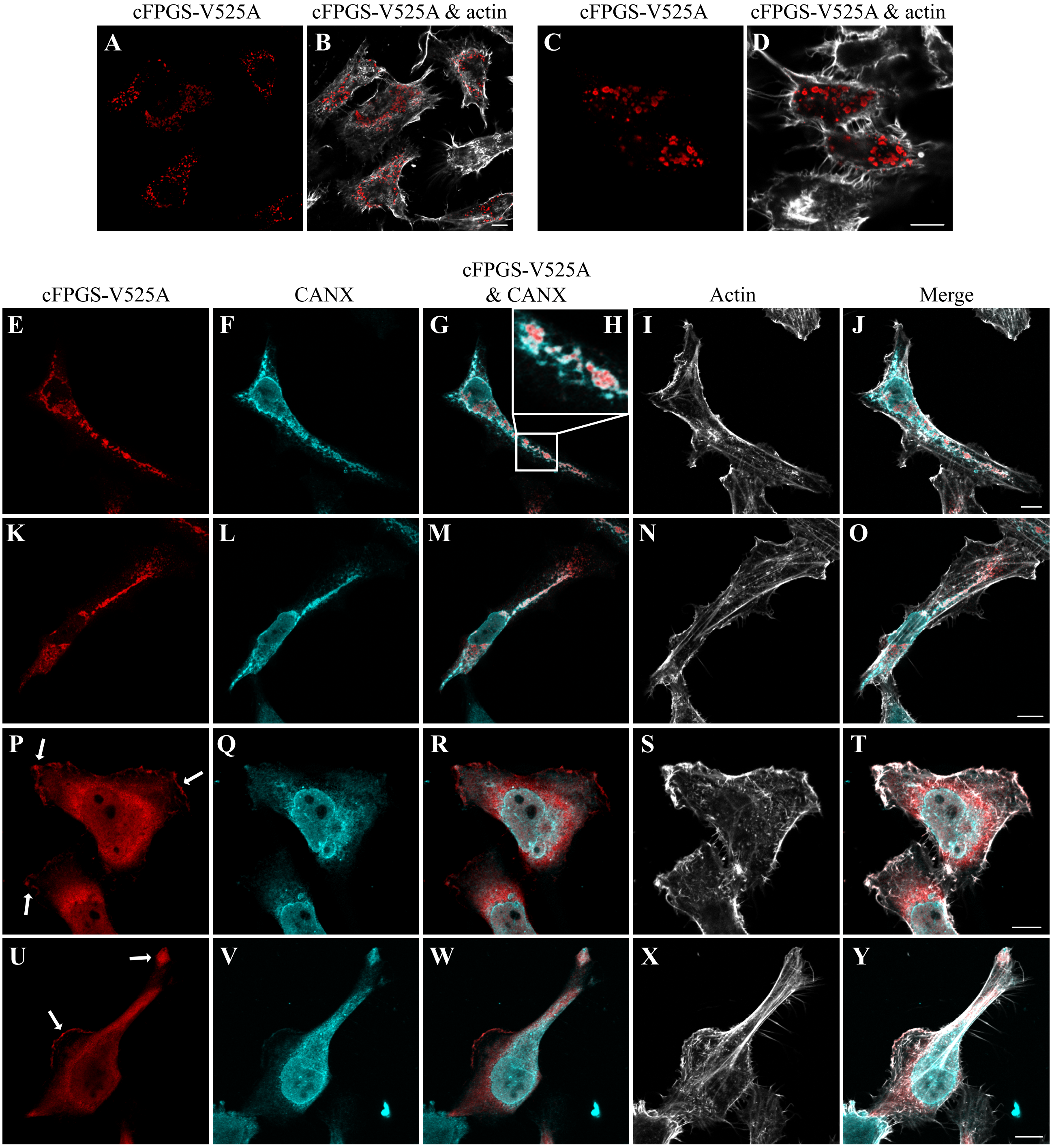

Figure S4: FA availability regulates the subcellular localization of FPGS translation. (A-D) HeLa cells, transfected with F-cFPGS-V525A under complete growth medium conditions. HeLa cells deprived of FA for 14 days, were transfected with F-cFPGS-V525A-3'UTR under FA-free conditions (E-O) and after a 15 min FA-pulse (P-Y). H) Enlarged image of the boxed area showing cFPGS positive vesicle coated by CANX. Following fixation, cells were reacted with antibodies against FLAG (cFPGS-V525A, red) and CANX (blue), and with DyLight 650 Phalloidin for F-actin staining (white). Cells were scanned using a confocal microscope (x63 magnitude). The scale bars denote 10 µm.

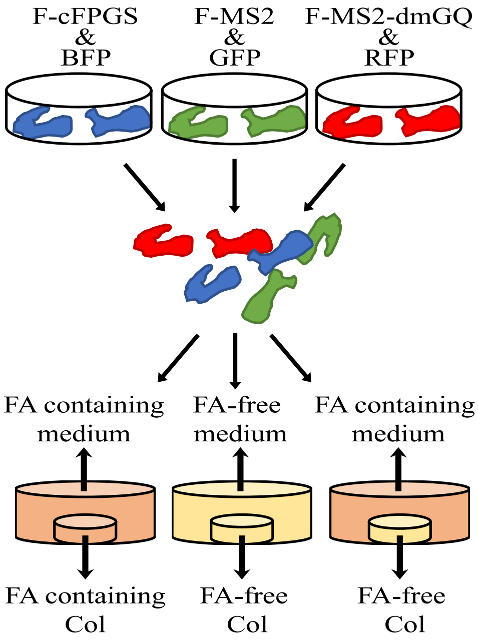

Figure S5: Design of cell migration assay under collagen-confined conditions. FA-deprived HeLa cells were co-transfected with either F-cFPGS & BFP, F-MS2 & GFP or F-MS2-dmGQ & RFP, mixed together at equal numbers and seeded on 35 mm glass-bottom plates. Cells were overlaid with collagen (Col) containing/lacking FA, and immersed in medium containing/lacking FA. Cells were incubated for 40 h before live fluorescence microscopy imaging.

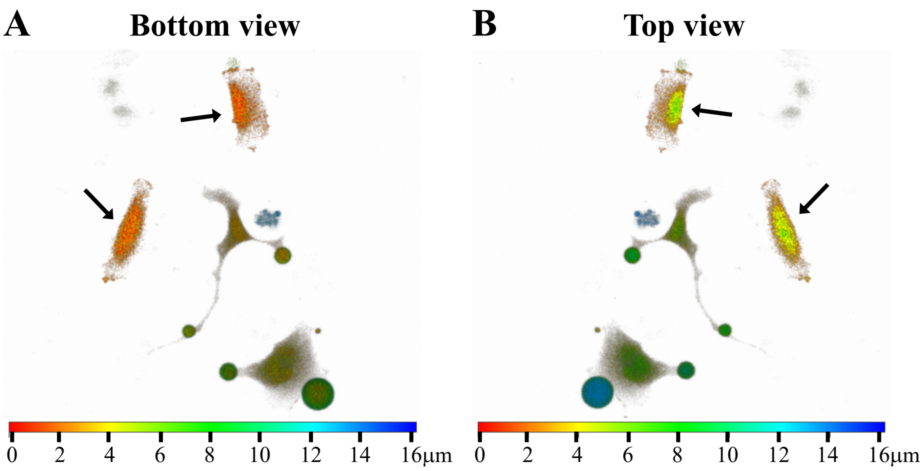

Figure S6: Disruption of the GQ sequences within the 3'UTR of FPGS impairs cell migration within Col gel matrix. Cells expressing F-MS2-dmGQ or F-MS2 overlaid with FA-free Col gel were supplemented with FA-containing medium. After 40 h, F-MS2-dmGQ expressing cells (arrows) retained their morphology and remained at the bottom of the gel layer, while F-MS2 expressing cells exhibited an amoeboid/mesenchymal hybrid migration phenotype. cells were visualized by confocal microscopy using focus stacking (i.e., Z-stacks), and 3D semi-translucent images were generated. The image is shown from a bottom (A) and top (B) view, with a color gradient representing the depth within the image.

Supplemental Movies legends

Movie S1: Transition through the Z-stacks of focus stacking imaging of F-cFPGS harboring cells in FA-containing collagen. FA-deprived HeLa cells, co-transfected with F-cFPGS (3'UTR-null) & BFP, were overlaid with FA-containing collagen and medium (2 µM). Following 40 h of incubation, cells were scanned by focus stacking with a confocal microscope at 1.5 µm intervals. Movie is shown at 1 fps.

Movie S2: Transition through the Z-stacks of focus stacking imaging of F-cFPGS harboring cells in FA-free collagen. FA-deprived HeLa cells, co-transfected with F-cFPGS (3'UTR-null) & BFP, were overlaid with FA-free collagen and medium. Following 40 h of incubation, cells were scanned by focus stacking with a confocal microscope at 1.5 µm intervals. Movie is shown at 1 fps.

Movie S3: Transition through the Z-stacks of focus stacking imaging of F-cFPGS harboring cells in collagen under a FA-gradient. FA-deprived HeLa cells, co-transfected with F-cFPGS (3'UTR-null) & BFP, were overlaid with FA-free collagen immersed in FA-containing medium (2 µM). Following 40 h of incubation, cells were scanned by focus stacking with a confocal microscope at 1.5 µm intervals. Movie is shown at 1 fps.

Movie S4: Transition through the Z-stacks of focus stacking imaging of F-MS2 harboring cells in FA-containing collagen. FA-deprived HeLa cells, co-transfected with F-MS2 (WT 3'UTR) & GFP, were overlaid with FA-containing collagen and medium (2 µM). Following 40 h of incubation, cells were scanned by focus stacking with a confocal microscope at 1.5 µm intervals. Movie is shown at 1 fps.

Movie S5: Transition through the Z-stacks of focus stacking imaging of F-MS2 harboring cells in FA-free collagen. FA-deprived HeLa cells, co-transfected with F-MS2 (WT 3'UTR) & GFP, were overlaid with FA-free collagen and medium. Following 40 h of incubation, cells were scanned by focus stacking with a confocal microscope at 1.5 µm intervals. Movie is shown at 1 fps.

Movie S6: Transition through the Z-stacks of focus stacking imaging of F-MS2 harboring cells in collagen under a FA-gradient. FA-deprived HeLa cells, co-transfected with F-MS2 (WT 3'UTR) & GFP, were overlaid with FA-free collagen immersed in FA-containing medium

(2 μM). Following 40 h of incubation, cells were scanned by focus stacking with a confocal microscope at 1.5 μm intervals. Movie is shown at 1 fps.

Movie S7: Transition through the Z-stacks of focus stacking imaging of F-MS2-dmGQ harboring cells in FA-containing collagen. FA-deprived HeLa cells, co-transfected with F-MS2dmGQ (GQ double mutant 3’UTR) & RFP, were overlaid with FA-containing collagen and medium (2 μM). Following 40 h of incubation, cells were scanned by focus stacking with a confocal microscope at 1.5 μm intervals. Movie is shown at 1 fps.

Movie S8: Transition through the Z-stacks of focus stacking imaging of F-MS2-dmGQ harboring cells in FA-free collagen. FA-deprived HeLa cells, co-transfected with F-MS2-dmGQ (GQ double mutant 3’UTR) & RFP, were overlaid with FA-free collagen and medium. Following 40 h of incubation, cells were scanned by focus stacking with a confocal microscope at 1.5 μm intervals. Movie is shown at 1 fps.

Movie S9: Transition through the Z-stacks of focus stacking imaging of F-MS2-dmGQ harboring cells in collagen under a FA-gradient. FA-deprived HeLa cells, co-transfected with F-MS2-dmGQ (GQ double mutant 3’UTR) & RFP, were overlaid with FA-free collagen immersed in FA-containing medium (2 μM). Following 40 h of incubation, cells were scanned by focus stacking with a confocal microscope at 1.5 μm intervals. Movie is shown at 1 fps.
